# Understanding heterogeneity in the pathogenesis and drug responses of ulcerative colitis through single-cell and spatial transcriptomics

**DOI:** 10.3389/fimmu.2026.1794207

**Published:** 2026-03-31

**Authors:** Jennifer Phillips, George Tambakis, Rina Kumar, Anthony Croft, Ian Brown, Christophe Rosty, Robert Anderson, Gabrielle Belz, Amanda J. Oliver, Albert Xiong, Quan Nguyen, Graham Radford-Smith, Gareth J. Walker

**Affiliations:** 1 Department of Gastroenterology and Hepatology, Inflammatory Bowel Disease Unit, Royal Brisbane and Women’s Hospital, Brisbane, QLD, Australia; 2 The Faculty of Medicine, University of Queensland, Brisbane, QLD, Australia; 3 Queensland Institute for Medical Research (QIMR) Berghofer, Brisbane, QLD, Australia; 4 Envoi Specialist Pathologists, Kelvin Grove, QLD, Australia; 5 Colorectal Oncogenomic Group, Department of Clinical Pathology, The University of Melbourne, Parkville, VIC, Australia; 6 Department of Gastroenterology, Mackay Base Hospital, Mackay, QLD, Australia; 7 The University of Queensland Frazer Institute, University of Queensland, Brisbane, QLD, Australia; 8 National Centre for Spatial Tissue and AI Research (NCSTAR), Brisbane, Australia

**Keywords:** inflammatory bowel disease, single-cell RNA sequencing, spatial proteomics, spatial transcriptomics, treatment response

## Abstract

**Introduction:**

Ulcerative colitis (UC) is driven by mucosal inflammation and epithelial injury. Single cell RNA sequencing (scRNA-seq) enables high resolution profiling of immune, stromal and epithelial compartments in UC, whilst spatial transcriptomic (ST) and proteomic (SP) enable interrogation of cell-cell interactions, niche-specific expression profiles, and spatially restricted pathological processes. Although scRNA-seq, ST, and SP technologies have been rapidly evolved in the past ten years, relatively limited clinical applications have been demonstrated. This systematic review aims at comprehensively analysing current evidence in UC studies as an approach to identifying key limitations and proposing recommendations for strengthening future spatial research towards translations into clinics.

**Methods:**

A comprehensive search of Embase, Medline, and grey literature was conducted to identify studies using scRNA-seq or spatial transcriptomic or proteomic technologies in adult UC cohorts. Outcomes of interest included insights into disease pathogenesis or treatment response.

**Results:**

scRNA-seq studies revealed alterations across the innate and adaptive immune systems, as well as stromal and epithelial compartments in UC colonic tissue. Spatial studies provided insights into: (i) cellular composition of the UC microenvironment; (ii) inflammatory features in treatment responders versus non-responders; and (iii) ligand-receptor interactions as potential spatial biomarkers and therapeutic targets.

**Conclusion:**

Overall, single-cell and spatial studies are deepening our understanding of UC pathogenesis and treatment response. However, they are often limited by small sample sizes and heterogeneous UC phenotypes. Future studies should prioritise robust cohort design and careful sample stratification. This will be critical to generating mechanistically precise, reproducible, and clinically meaningful insights into the heterogeneity of UC pathogenesis and treatment response.

**Systematic Review Registration:**

https://www.crd.york.ac.uk/PROSPERO/, identifier CRD42024601628.

## 1 Introduction

Ulcerative colitis (UC) is a chronic and debilitating form of Inflammatory Bowel Disease (IBD) with a rising global incidence (1). While it’s aetiology remains incompletely understood, UC is thought to arise from dysregulated immune, stromal and epithelial responses to environmental and microbial stimuli in genetically susceptible individuals, ultimately leading to mucosal inflammation and epithelial injury (2, 3). The disease’s pathogenesis involves a complex interplay between immune and non-immune cells, genetics, microbiome, nutrients and metabolites, contributing to its marked clinical heterogeneity (4).

Recent advances in IBD treatment have led to an expanding range of biologic and small molecule advanced therapies. Management has shifted from broad-spectrum immunosuppressants, such as corticosteroids and thiopurines, towards more targeted therapies including biologics (for example anti-TNFs, anti-IL12/23, anti-IL23s and anti-integrins) and small molecules such as Janus kinase inhibitors (5). Despite these advances, only 50-60% of patients who maintain follow up with a treating physician achieve a sustained treatment response, highlighting the urgent need for a precision medicine approach in IBD (6, 7).

Traditional disease assessment methods, including endoscopic and histological evaluation, remain essential for assessing disease severity. However, they provide limited insights into underlying disease mechanisms and are insufficient for guiding individualised treatment decisions (8, 9).

To better understand the cellular and molecular mechanisms underlying IBD pathogenesis and treatment response, early human studies relied on bulk tissue analysis from the gastrointestinal tract. These studies used a range of transcriptomic and proteomic approaches, including microarrays, bulk RNA sequencing and mass spectrometry–based proteomics, to characterise molecular alterations associated with disease (10–12). However, due to the inherent heterogeneity of intestinal tissue and the diversity of immune cell populations, these bulk approaches often failed to resolve cell type–specific transcriptional signatures, instead predominantly reflecting signals from the most abundant cell types and highly expressed genes (13).

The advent of single-cell technologies helped overcome some of these limitations by revealing previously unrecognised cellular diversity within each of the intestinal epithelium, stroma, and immune compartments (14). Yet, these approaches lacked spatial context, making it difficult to determine how distinct cell populations are organised and interact within the tissue microenvironment (15). Spatial transcriptomics now bridges this gap by preserving the tissue architecture and combining gene expression profiling with spatial localisation. This technology enables the mapping of gene expression patterns in intact tissue sections offering critical insights into the spatial distribution and interaction of specific cell types and molecular pathways *in situ* (16). Spatial proteomics extends the insights gained from spatial transcriptomics by linking gene expression patterns to protein function, thereby deepening our understanding of cellular interactions within their native tissue context. Although the application of spatial methods to IBD research is in its early stages, these technologies hold promise for elucidating disease pathogenesis mechanisms, refining drug-target interactions and identifying novel biomarkers and therapeutic targets in UC (17).

While these technologies offer powerful new insights, they also produce large and complex datasets, with relatively limited clinical applications. To ensure reliable and clinically meaningful results, it is essential to replicate findings and identify consistent patterns across studies, enabling robust and generalisable conclusions. However, current literature is hindered by limited reproducibility of key findings and a lack of precision in cohort design and clinical metadata collection. This review aims to synthesise current evidence from single-cell transcriptomic, spatial transcriptomic, and spatial proteomic studies as they relate to ulcerative colitis (UC) pathogenesis and treatment response. We also highlight existing gaps and limitations in the field and propose recommendations to inform and strengthen future research.

## 2 A comprehensive review framework from a clinical perspective

### 2.1 Data collection

We performed a systematic review following PRISMA guidelines, prospectively registered with the PROSPERO database (registration ID: CRD42024601628). A comprehensive literature search was performed using PubMed and Embase from inception to December 1, 2025 (**Supplementary Figure 1**). A search for grey literature was performed using Google Scholar. The search strategy included the keywords *single-cell gene expression analysis*, sp*atial analysis* and *ulcerative colitis*. Additionally, reference lists of included studies and relevant conference abstracts were manually searched.

Studies were eligible for inclusion if they were peer-reviewed, published in English and directly investigated the application of single cell RNA sequencing (scRNA-seq) or spatial transcriptomics or proteomics, or a combination of these methods, in adult patients with UC. Only studies analysing colonic mucosal samples were included. Studies utilising data from the Gene Expression Omnibus (GEO) database were excluded unless experimental validation at single-cell resolution was performed. Both spatial transcriptomics and proteomics studies were included and given the limited research on spatial technologies in UC to date, relevant conference abstracts were also considered.

The outcomes of interest were insights into UC pathogenesis and treatment response. Studies focusing on colitis-associated cancer as well as ileal pouch-anal anastomosis and pouchitis were excluded from analysis. Main clinically relevant data were extracted, including study population characteristics (number of UC patients, clinical characteristics, details on samples collected and inclusion of a healthy control group), and key outcomes (differential gene expression by cell type, cellular neighbourhood interactions, findings related to treatment response or non-response etc.). Due to the heterogeneity of methodologies and outcome measures across studies, a formal meta-analysis was not performed.

To assess the risk of bias, a customized tool was developed based on the seven domains of bias outlined in the ROBINS-I tool, as no standardized risk-of-bias framework exists for *in vitro* studies. Conventional tools, such as the ROBINS-I and Cochrane risk-of-bias framework were developed for clinical interventional studies and do not capture methodological issues specific to single-cell and spatial -omics research. The customised risk-of-bias assessment tool used in this review is provided in **Supplementary Table 1** (18).

### 2.2 Classifying studies to interpret clinical heterogeneity

The studies included in this systematic review employ robust scientific methodologies; however, they are often limited by small sample sizes and heterogeneous UC phenotypes. A summary of the clinical phenotyping data for samples used in the scRNA-seq and spatial analyses is provided in **Table 1**. To help interpret the clinical heterogeneity of included samples, and its impact on the reliability and generalisability of study findings, we suggest that studies can be broadly categorised into three groups:

**Table 1:** Summary of the clinical data from the samples used in each study.

Study Author / *Journal*	HC	UC	UC disease distribution	UC time since diagnosis	UC disease activity	UC treatment	Site of biopsies	Non-inflamed and inflamed biopsies
**Boland et al.** (30) *Science Immunology, 2020*	9	7	E1 n=0 E2 n=1 E3 n=6	8.3yrs +/- 6.8yrs	Mild n=1 Moderate n=5 Severe n=1	5ASA n=4 Thiopurine n=1 Vedo n=1 Tofa n=1	Rectum	No
**Chen et al.** (45) *Gastroenterology, 2021*	0	4	E1 n=3 E2 n=0 E3 n=1	Not reported	Mild n=2 Moderate n=1 Severe n=1	None n=2 5ASA n=2	Rectum (inflamed) Sigmoid (uninflamed)	Yes
**Corridoni et al.** (40) *Nature Medicine, 2020*	3	3	E1 n=0 E2 n=3 E3 n=0	Not reported	Mild n=1 Moderate n=2 Severe n=0	5ASA topically n=1 5ASA oral n=1 Thiopurine n=1	Distally inflamed colon	No
**Kinchen et al.** (46) *Cell, 2018*	5	5	Not reported	Newly diagnosed	Not reported	No medical therapies	Not reported	3/5
**Korsunsky et al.** (47) *Med, 2022*	5	8	Not reported	Not reported	Mild n =1 Moderate n=2 Severe n=4 Unknown n=1	5ASA n=4 Thiopurine n=1 MTX n=1 Vedo n=1 Unknown n=1	Rectum Sigmoid Descending colon	4/8 *inf and adj not inf but same Nancy Index reported
**Li et al.** (37) *Cellular and Molecular Gastroenterology and Hepatology, 2021*	4	5	E1 n=0 E2 n=5 E3 n=0	3-18years	Mild n=0 Moderate n=5 Severe n=0	No treatment for past 3 months	Inflamed sigmoid Non-inflamed ascending	Yes
**Luo et al.** (33) *Frontiers in Molecular Biosciences, 2022*	2	4	Not reported	Not reported	Not reported	Not reported	Not reported	Not reported
**Mitsialis et al.** (25) *Gastroenterology, 2020*	18	5	E1 n=0 E2 n=0 E3 = 5	0-15years	Mild n=1 Moderate n=1 Severe n=3	Immunomodulator n=2 Anti-TNF n=1 Anti-TNF + IM n=1 Steroid n=2 (IM pts) No treatment n=1	Transverse colon n=4 Left colon n=1	No
**Mo et al.** (48) *American Journal of Human Genetics, 2021*	0	4	Not reported	Not reported	Not reported	Not reported	Rectum (inflamed) and Sigmoid (uninflamed)	Yes
**Parikh et al.** (41) *Nature, 2019*	3	3	Not reported	Not reported	Not reported	Immunotherapy naïve	Distal (inflamed), Proximal (non-inflamed)	Yes
**Scheid et al.** (31) *Journal of Experimental Medicine, 2023*	8	13	Not reported	5-25years	Inflamed Mild n=2 Moderate n=1 Severe n=5 Non-inflamed Remission n=3 Mild n=2	Topical 5ASA n=1 Thiopurine n=1 Vedo n=2 Tofa n=3 IFX n=3 ADA n=2 Rizankizumab n=1	Not reported	Yes 8x active 5x remission
**Smillie et al.** (22) *Cell, 2019*	12	18	Not reported	Not reported	Not reported	Not reported	Rectum n=7 Sigmoid n=5 Left colon n=1 Transverse n=2 Right colon n=2 Unknown n=1	Yes
**Uzzan et al.** (32) *Nature Medicine, 2022*	5	4	E1 n=0 E2 n=2 E3 n=2	0–42 years	Mild n=0 Moderate n=2 Severe n=2	None n=1 5ASA n=3	Left colon	No
**Zhou et al.** (24) *Journal of Translational Medicine, 2025*	0	7	Not reported	0–5 years	Mild n=0 Moderate n=5 Severe n=2	5ASA n=7	Not reported	Yes
**Friedrich et al.** (50) *Nature Medicine, 2021*	4	7	Not reported	4-62years	Mild n=1 Moderate n=2 Severe n=4	Not reported	Not reported	No
**Hsu et al.** (51) *Inflammatory Bowel Diseases, 2023*	0	13	E1 n=2 E2 n=3 E3 n=8	Not reported	Remission n=3 Mild n=4 Moderate n=3 Severe n=3	All vedolizumab Concomitant Rx MTX n=1 Thiopruine n=1, Pred n=1 Prior treatments 5ASA n= 6 ADA n=2 IFX n=2 IFX and ADA n=3	Not reported	No
**Du et al.** (36) *Nature Communications, 2023*	scRNAseq 4	scRNAseq 4	Not reported	3-18years	Mild n=0 Moderate n=4 Severe n=0	Not reported	Not reported	Not reported
	Spatial 19	Spatial 33	E1 n=8 E2 n=17 E3 n=8	Not reported	Remission n=1 Mild n=8 Moderate n=13 Severe n=11	Not reported	Not reported	Not reported
**Garrido-Trigo et al.** (49) *Nature Communications, 2023*	scRNAseq 6	scRNAseq 6	E1 n=2 E2 n=3 E3 n=1	3-25years	Mild n=0 Moderate n=0 Severe 3 n=6	5ASA n=2 Thiopurine n=1 Prednisolone n=1 Vedolizumab n=2	Rectum n=2 Sigmoid n=3 Transverse n=1	No
	Spatial 3	Spatial 3	E1 n=0 E2 n=0 E3 n=3	5–21 years	Mild n=0 Moderate n=0 Severe n=3	IFX n=1 Tofacitinib n=2	Sigmoid n=2 Transverse n=1	No
**Jha et al.** (26) *BioRχiv, 2023*	scRNAseq 5	scRNAseq 9	E1 n=0 E2 n=3 E3 n=6	Not reported	Mild n=1 Moderate n=2 Severe n=6	5ASA n=1 5ASA plus IV steroid n=1 Topical steroid n=1 Infliximab n=2 Adalimumab plus IV steroid n=1 IV steroid n=2 Not reported n=1	Not reported	No
	Spatial 2	Spatial 2	E1 n=0 E2 n=0 E3 n=2	Not reported	Mild n=0 Moderate n=0 Severe n=2	Prednisolone + 5ASA n=1 Budesonide + 5ASA n=1	Not reported	No
**Mennillo et al.** (3) *Nature Communications, 2024*	scRNAseq 4	scRNAseq 8	E1 n=1 E2 n=5 E3 n=2	5–25 years	Mild n=5 Moderate n=2 Severe n=3	5ASA n=4 VDZ n=4	Left colon n=1 Left and right colon n=7	No
	Spatial 4	Spatial 8	E1 n=1 E2 n=5 E3 n=2	5–25 years	Mild n=5 Moderate n=2 Severe n=3	5ASA n=4 VDZ n=4	Left colon n=1 Left and right colon n=7	No
**Thomas et al.** (23) *Nature Immunology, 2024*	scRNAseq 3	scRNAseq 22	E1 n=3 E2 n=9 E3 n= 10	6 years +/- 5 years	Mild n=0 Moderate n=7 Severe n=15	Biologic naïve, further detail not reported	Throughout colon, rectum and TI	No (post-Rx biopsies were site-matched)
	Spatial 3	Spatial 22	E1 n=3 E2 n=9 E3 n= 10	6 years +/- 5 years	Mild n=0 Moderate n=7 Severe n=15	Biologic naïve, further detail not reported	Throughout colon, rectum and TI	No (post-Rx biopsies were site-matched)
**Lafzi et al.** (17) *Molecular Systems Biology, 2024*	4	2	Not reported	Not reported	Not reported	Not reported	Not reported	No
**Lyu et al.** (69) *Frontiers in Cell and Developmental Biology, 2022*	7	8	E1 n=2 E2 n=3 E3 n=3	1.6+/-2.7years	MES 2.3 +/-0.8	None n=2 5ASA n=5 5ASA plus steroid n=1	Sigmoid n=2 Rectum n=6	No
**vanUnen et al.** (52) *Frontiers in Immunology, 2022*	4	4	Not reported (for spatial)	Not reported (for spatial)	Not reported (for spatial)	All treatment naïve	Not reported (for spatial)	Yes
**Venkat et al.** (53) *United European Gastroenterology Jornal, 2025*	0	45	Not reported	Not reported	Mild n=16 Moderate n=0 Severe n=29	Not reported	Not reported	No
Zhang et al.* (59) *Journal of Crohn’s and Colitis, 2024*	2	4	Not reported	Not reported	Not reported	Not reported	Not reported	Not reported
Holman et al.* (55) *Journal of Crohn’s and Colitis, 2025*	5	29	Not reported	Not reported	Not reported	Not reported	Not reported	Not reported
Kim 2024* (56) *Journal of Crohn’s and Colitis*	2	54	Not reported	Not reported	Not reported	TNF therapy as primary biologic	Not reported	No (post-Rx biopsies were site-matched)
**Mayer et al.** (54) *Science Advances, 2023*	5	29	Not reported	8.86+/-10.8 years and 7.2+/-5.9 years	Mild n=4 Moderate n=13 Severe n=12	TNF therapy n=29	Rectum n=24 Left colon n=2 Right colon n=3	Not reported

* abstract only.

- Tissue atlas studies: These aim to create a comprehensive cellular map of ulcerative colitis. In these studies, clinical heterogeneity may be advantageous, offering a broader view of disease biology, provided the sample size is sufficient, and a range of well-characterised UC phenotypes are adequately represented.
- Hypothesis-driven studies: These investigate specific clinical questions, such as identifying transcriptional features associated with treatment response. In this context, clinical heterogeneity may limit the generalisability of findings unless results are validated in an independent cohort with consistent clinical characteristics.
- Small, heterogeneous studies: These include a limited number of samples with poorly defined or unevenly distributed phenotypes. Such studies may lack the statistical power and representativeness needed to draw meaningful conclusions about the broader UC population, but may contribute as proof of feasibility or methodology development.

The authors note that emerging research is generating integrated atlases by combining individual single-cell RNA sequencing studies into large-scale datasets. These atlases enhance the power of individual studies and enable comparative analyses. One key advantage is the ability to detect rare but important cell types that may otherwise be obscured within larger clusters (19).

When presenting results, to provide a more reliable overview of the scRNA-seq findings, we focus primarily on results from the larger, tissue atlas studies, highlighting instances where results from smaller studies may contribute by supporting and validating these observations, or report an opposite trend. When presenting our findings related to treatment-response we will highlight where clinical heterogeneity may limit generalisability of findings to the broader UC population.

### 2.3 Spatial transcriptomic and proteomic analysis

Recent advances in single-cell technologies have transformed the study of complex tissues by enabling high-throughput profiling of gene expression at the resolution of individual cells. Droplet-based platforms now allow the simultaneous analysis of thousands to hundreds of thousands of cells, facilitating the identification of rare cell populations and previously unrecognised cellular heterogeneity (13). In parallel, the development of single-cell multi-omic approaches, which combine measurements of gene expression with other molecular layers such as chromatin accessibility or surface protein expression, has further expanded the ability to define cellular states and regulatory networks (13, 14). Advances in computational methods have also enabled the integration of large single-cell datasets and the construction of detailed cellular atlases of human tissues (19).

Spatial transcriptomic and proteomic technologies now allow these cellular insights to be examined within intact tissue architecture. An overview of the spatial technologies and their key characteristics is provided in **Supplementary Table 2**.

Spatial transcriptomic technologies in general, involve a trade-off between cellular resolution and transcriptomic breadth: approaches offering single-cell resolution typically rely on targeted panels comprising several thousand genes, whereas methods enabling whole-transcriptome analysis generally do so at the expense of confident single-cell resolution. Broadly, these platforms can be classified into two main categories:

- Sequencing-based methods use spatially barcoded arrays to capture RNA from tissue sections. After tissue lysis and RNA capture, transcripts or probes are sequenced and computationally mapped back to their original spatial coordinates. These methods traditionally offered a resolution of ~50–100 µm, meaning that each spot captures RNA from multiple cells, limiting single-cell resolution. However, in the past few years, ST technologies have been developed to produce single-cell resolution data (e.g. Stereoseq, Visium HD and Curio).
- Imaging-based methods rely on high-resolution fluorescence microscopy combined with *in situ* hybridization to directly detect and localize specific RNA molecules. These methods, such as MERFISH and seqFISH, Xenium and CosMx achieve single-cell or even subcellular resolution but are usually limited to detecting a targeted set of transcripts, rather than the entire transcriptome. However, these methods are expanding and are now able to include up to 5000 genes.

Spatial proteomic technologies, in contrast, map the spatial distribution of proteins within tissues, and typically use either imaging-based or mass spectrometry-based technologies:

- Imaging-based methods use multiplexed antibody staining with fluorophore or metal-conjugated tags. Technologies such as CODEX (CO-Detection by Indexing), Imaging Mass Cytometry (IMC), and Multiplexed Ion Beam Imaging (MIBI) allow simultaneous detection of dozens of proteins with high spatial resolution. CODEX, for example, uses repeated cycles of staining and imaging with DNA-barcoded antibodies to build highly multiplexed protein maps. While the theoretical upper limit of protein detection is around 60 markers, most studies typically use panels of 20–30 proteins.
- Mass spectrometry-based methods (e.g., MALDI Imaging, Hyperion) detect proteins or peptides directly from tissue sections without the need for antibodies. These methods offer broader detection of the proteome but typically at lower spatial resolution compared to imaging-based techniques.

Together, spatial transcriptomic and proteomic technologies offer complementary insights into the mechanisms driving UC pathogenesis and treatment response by enabling the exploration of key spatial features, including:

- Cell-Type Mapping: Identifies and localises distinct cell populations within tissue, revealing how their spatial arrangement contributes to disease processes.
- Cell-Cell Interactions: Infers intercellular communication by identifying ligand–receptor pairs expressed in neighbouring cells, helping uncover signalling networks underlying inflammatory pathways and treatment response.
- Neighbourhood Analysis: Uses spatial clustering to detect microenvironments, such as immune aggregates or fibrotic zones, defined by similar gene or protein expression profiles.
- Spatial Co-Expression Networks: Highlights gene modules or pathways (e.g., Wnt, Notch) that are co-expressed in specific tissue regions, revealing spatially regulated biological processes (20).

### 2.4 A clinically relevant set of criteria to assess works in IBD

Spatial transcriptomics and proteomics hold great promise for advancing our understanding of IBD. However, these technologies produce vast amounts of data so must be applied thoughtfully, and with clinical relevance, to truly impact the field. Drawing on lessons from their application in oncology, we propose the following three key aims to guide their use in the field of IBD (21):

1. To resolve the cellular composition of the UC microenvironment and map its spatial organization.
2. To integrate cellular composition analysis with spatial co-localization to distinguish inflammatory microenvironments across drug response subtypes.
3. To utilize spatially defined ligand-receptor interactions as potential biomarkers for predicting drug response and identify novel therapeutic targets.

By reviewing how the included studies address these aims, we seek to determine how the current literature answers important clinical questions, identify existing knowledge gaps, and suggest directions for future research.

**Figure 1:**
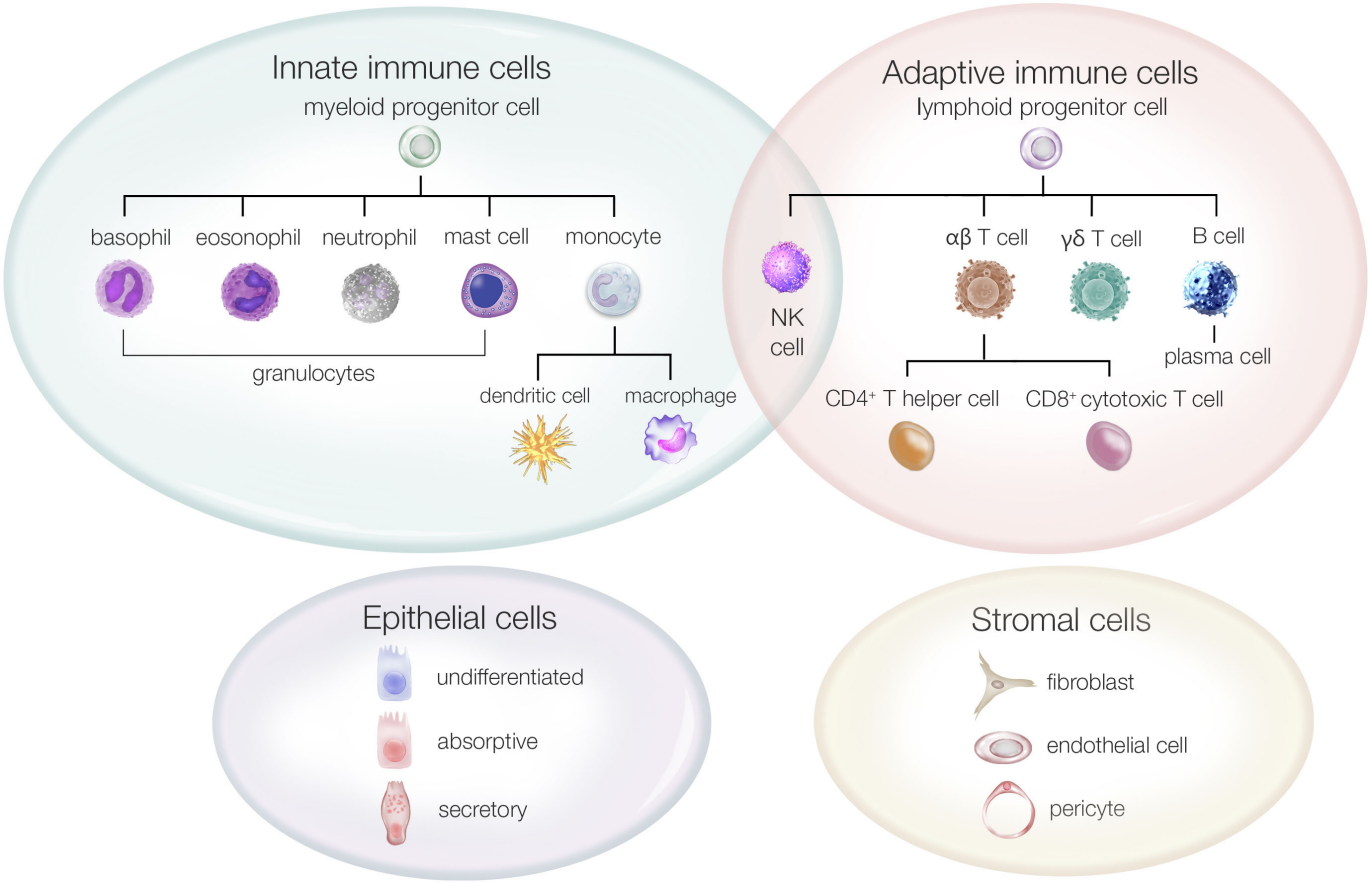
Main intestinal cell populations in the human colon.

**Figure 2:**
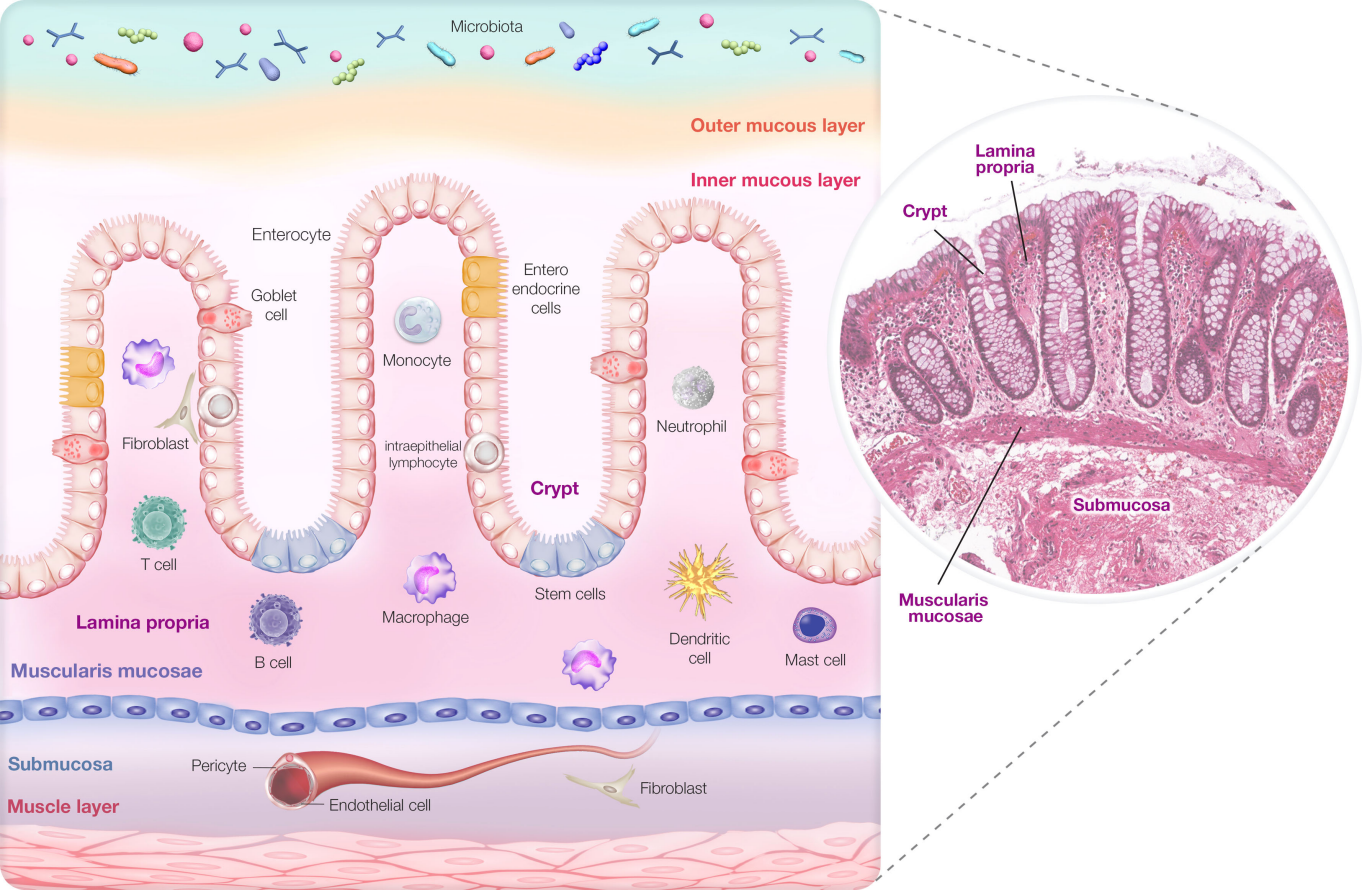
Diagram of the colonic mucosal anatomy.

To support interpretation of the results, we provide an overview of the major intestinal cell populations (**Figure 1**) and a schematic diagram of the intestinal mucosal anatomy (**Figure 2**).

## 3 Key biological insights relevant to pathogenesis and treatment responses of UC from scRNA-seq studies

The systematic search of databases and other sources identified 526 records for review. After title and abstract screening, followed by full text review, 29 studies met the inclusion criteria (**Supplementary Figure 1**).

Of the included studies 16 studies utilised single-cell RNA sequencing (scRNA-seq), 8 studies utilised spatial transcriptomic and/or proteomic techniques, and 5 incorporated both methodological approaches. Twenty studies focused on the pathogenesis of UC, 5 specifically examined treatment response, 3 addressed both pathogenesis and treatment response, and one used single-cell data to develop a transcriptional risk score for predicting colectomy in UC. Among the 8 studies assessing treatment response, 6 utilised spatial techniques. Study characteristics and results are summarised in **Table 2**.

**Table 2:** Study characteristics and key findings.

Study ID	Method of analysis	Study population	Study outcomes	Description of study findings
Boland 2020 (30)	scRNA-seq	UC: 7 HC:9	Pathogenesis	Plasma cells o Increased IgG1+ plasma cells in UC colonic tissue. T cells o Regulatory T cells showed altered transcriptional program with ↑ SATB1, ZEB2 and ↓ KLF2, MYC, ITGB1. o Three distinct subclusters of γδ T cells identified with expression of CCR7, KLRB1, GNLY, XCL1. o Clonally related CD8+ tissue-resident memory T cells shifted toward an inflammatory phenotype, associated with ↑ EOMES.
Chen 2021 (45)	scRNA-seq	UC: 4 HC: 0	Pathogenesis	Mast cells o Activated mast cells enriched in UC with increased expression of CPA3 and TPSAB1. Fibroblasts / epithelial cells o ADM expression detected in activated fibroblasts and secretory epithelial cells.
Corridoni 2020 (40)	scRNA-seq	UC: 3 HC:3	Pathogenesis	T cells o Tissue-resident memory, effector and intraepithelial T cells showed altered activation signatures with ↓ SPINK2, FOS, CD160 and ↑ TNFRSF9, CTLA4. o IL26+ T cells / IELs o Enriched expression of KIR3DL2, IL26, IL23R. Epithelial o Multiple epithelial subpopulations showed increased expression of HLA-E. Cell–cell signalling o Ligand–receptor interactions included IL18–IL18R1/IL18RAP and TNF–TNFRSF1A signalling between epithelial and CD8+ T cells.
Kinchen 2018 (46)	scRNA-seq	UC: 5 HC:5	Pathogenesis	Stromal o Identification of a niche stromal population near epithelial crypts expressing SOX6, F3 and WNT genes. o Inflammatory stromal cells o Expanded stromal population expressing CCL19, CCL21, IL33 and TNFSF14
Korsunsky 2022 (47)	scRNA-seq	UC: 8 HC:5	Pathogenesis	Fibroblasts o Seven fibroblast subsets identified including WNT2B+ crypt-associated fibroblasts, RSPO3+ stem-cell niche fibroblasts, and inflammatory fibroblasts. o CXCL10+CCL19+ immune-interacting fibroblasts and SPARC+COL3A1+ vascular-interacting fibroblasts were expanded in inflamed tissue.
Li 2021 (37)	scRNA-seq	UC: 5 HC:4	Pathogenesis	Plasma cells o Two plasma cell subsets enriched in UC: MZB1+ plasma cells and IGLL5+ plasma cells. T cells o Increased signalling genes including FYN, PTPRC and STAT3 in T-cell populations. Epithelial o Reduced abundance of TRMP5+ tuft cells. o Epithelial cells showed increased expression of antigen-presentation genes including HLA-DQA1, HLA-DQB1, HLA-DRB1.
Luo 2022 (33)	scRNA-seq	UC: 4 HC:2	Pathogenesis	T cells o Enrichment of CCR6+TNF+CD161+ effector memory T cells in active UC. Myeloid o Increased inflammatory macrophages/monocytes expressing HLA-DR, CD14, IL21. Other immune populations o Increased CXCR3+CCR4+ naïve B cells, cytotoxic NK cells (IFNG+), and activated dendritic cells.
Mitsialis 2020 (25)	scRNA-seq	UC: 5 HC:18	Pathogenesis	T cells o Expansion of IL17A+CD161+ effector memory T cells and IL17A+ regulatory T cells. Myeloid o Myeloid cluster expressing IL1B, CD14 and FCGR3A enriched in UC. Granulocytes o Increased HLA-DR+CD56+ granulocytes. Innate lymphoid cells o Reduced ILC3 populations
Mo 2021 (48)	scRNA-seq	UC: 4 HC:0	Other	T cells o Increased expression of TNFRSF4 and TNFRSF18 in immunoregulatory T cells. Innate immune cells o CDC42SE2 expression in ILC3 populations. o Increased PTK2B (macrophages Stromal/endothelial cells o Increased expression of PDGFB (endothelial cells), and MRPL20 (fibroblasts).
Parikh 2019 (41)	scRNA-seq	UC: 3 HC:3	Pathogenesis	Epithelial cells o Downregulation of metabolic pathways with increased antimicrobial gene expression including SAA1, DMBT1, PLA2G2A. o BEST4+ epithelial cells showed Reduced metallothionein gene expression. o Goblet cells showed disrupted transcriptional programs with ↑ LYZ and ↓ WFDC2. o Reduced HB-EGF signalling impacting Wnt/β-catenin pathways.
Scheid 2023 (31)	scRNA-seq	UC: 13 HC:8	Pathogenesis	Plasma cells o Shift from IgA-dominant to IgG1-dominant plasma cells in UC. o Increased expression of antibody production and ER-stress genes including MZB1, XBP1 and CD79A/B.
Smillie 2019 (22)	scRNA-seq	UC: 18 HC:12	Pathogenesis	T cells o Increased CD8+IL17+ T cells and regulatory T cells. Myeloid o Increased inflammatory monocytes. Epithelial o Identification of BEST4+ colonocytes, a distinct epithelial cell type expressing OTOP2/OTOP3. Stromal o Expansion of inflammation-associated fibroblasts (IAFs) expressing IL11, IL24, IL13RA2, FAP, TWIST1 and WNT2. Inflammatory signalling o OSMR expressed in fibroblasts and OSM enriched in inflammatory monocytes and DC2 cells
Uzzan 2022 (32)	scRNA-seq	UC: 4 HC:5	Pathogenesis	B cells o Expansion of naïve B cells with increased interferon signalling. Plasma cells o Increased proportion of IgG+ plasma cells. T cells o Expansion of CXCL13+ peripheral helper T cells expressing CD200, BCL6, TIGIT and ICOS. Macrophages o Inflammatory macrophages enriched in NF-κB, IL23 and TNF signalling genes
Zhou 2025 (24)	scRNA-seq	UC: 7 HC: 0	Pathogenesis	Macrophages o Distinct macrophage clusters between inflamed and non-inflamed UC tissue. o Increased FCN1+ and SPP1+ inflammatory macrophages with expression of MMP genes, CXCL5 and CXCL1. Fibroblasts o Increased CHI3L1+ fibroblasts expressing MMP genes and CXCL8.
Friedrich 2021 (50)	scRNA-seq	UC: 7 HC:4	Pathogenesis; Treatment response	Stromal o Increased inflammatory fibroblasts, pericytes and endothelial cells with reduced ABCA8+ and PDGFRA+ fibroblasts. o High expression of CXCL1, CXCL2, CXCL3, CXCL5 and CXCL8 within inflammatory fibroblasts Neutrophils o Neutrophil-associated inflammation o M4/M5 neutrophil signature associated with treatment non-response and elevated IL1B signalling.
Hsu 2023 (51)	scRNA-seq	UC: 13 (6 responders VDZ, 7 non-responders VDZ) HC:0	Treatment response	Vedolizumab response o Responders: Reduced Th17 cell proportions and increased IL2RB expression. o Non-responders: Th17 cells showed increased inflammatory transcriptional programs including IL17A, IL17F and IFNG. Increased inflammatory monocytes/macrophages expressing IL1A, IL1RN, OSM and CCL20.
Du 2023 (36)	scRNA-seq; Spatial proteomics *(Fluidigm Hyperion)*	scRNA-seq UC: 4 Spatial UC: 33 scRNA-seq HC:4 Spatial HC: 19	Pathogenesis	T cells o Increased regulatory T cells. Macrophages o Loss of tissue-resident macrophages with expansion of CD68+CCR2+ infiltrating macrophages expressing IL1B. Spatial organisation o Cellular neighbourhood analysis identified T-cell enriched, macrophage-centred and immunosuppressive niches in UC tissue.
Garrido-Trigo 2023 (49)	scRNA-seq; Spatial transcriptomics *(Nanostring CosMx SMI)*	scRNA-seq UC: 6 Spatial U: 6 scRNA-seq HC: 6 Spatial HC: 6	Pathogenesis	Macrophages o Increased macrophage heterogeneity with identification of inflammation-dependent alternative (IDA) macrophages expressing NRG1. Neutrophils o Three neutrophil states identified including IFN-responsive neutrophils (GBP1, IRF1). Spatial localisation o NRG1hi IDA macrophages localised to the subepithelial mucosa. o Strong spatial correlation between IDA macrophages and inflammatory fibroblasts.
Jha 2023 (26)	scRNA-seq; Spatial transcriptomics *(10x Genomics Visium)*	scRNA-seq UC: 9 Spatial UC: 2 scRNA-seq HC: 5 Spatial HC: 2	Pathogenesis; Treatment response	Cell population changes o Increased myeloid cells, plasma cells, mast cells, T cells and stromal cells, with reduced epithelial cells and innate lymphoid cells. o Increased Th17 cells in severe UC. Spatial organisation o Increased proximity between myeloid cells and epithelial cells, and between myeloid and T cells. o IgG+ plasma cells enriched in both epithelial and lamina propria compartments. Inflammatory drivers o Myeloid inflammatory genes including S100A8, S100A9, CXCL1 and TREM1 associated with histological activity.
Mennillo 2024 (3)	scRNA-seq; Spatial transcriptomics and proteomics *(IonPath MIBI, Akoya Biosciences CODEX, Nanostring CosMx SMI)*	scRNA-seq UC:8 (4 5ASA, 4 VDZ) Spatial UC: 8 (4 5ASA, 4 VDZ) scRNA-seq HC: 4 Spatial HC: 4	Treatment response	Cell population changes (UC vs HC) o Inflamed UC tissue showed increased Tregs, S2 fibroblasts, pericytes and endothelial cells, with reduced innate lymphoid cells and activated/memory CD4+ T cells. Fibroblasts o Activated fibroblasts expressed TIMP1, MMP1, MMP3 and AREG, while S2 fibroblasts were enriched for F3, POSTN, CXCL14 and PDGFRA. Myeloid and endothelial inflammation o Myeloid populations showed increased TIMP1, SOD2 and TYMP, while endothelial cells expressed TIMP1, MGP and S100A6, indicating coordinated stromal–immune activation. Spatial organisation o Activated fibroblasts and inflammatory myeloid cells were found in closer spatial proximity in UC, suggesting stromal–myeloid inflammatory niches. Vedolizumab response o Responders: higher epithelial repair genes (REG1A, OLFM4). o Non-responders: higher stromal/myeloid genes (MMP1, MMP2, THBS1).
Thomas 2024 (23)	scRNA-seq; Spatial transcriptomics and proteomics *(Nanostring GeoMx DSP)*	scRNA-seq UC: 22 (all Rx adalimmab) Spatial UC: 22 (all Rx adalimumab) scRNA-seq HC: 3 Spatial HC: 3	Treatment response	Plasma cells o Expanded IgG+ plasma cells in inflamed tissue. T cells o CXCL13+ peripheral helper T cells expressing checkpoint genes including PDCD1. Myeloid o Inflammatory monocytes expressing S100A8/9, TNF and IL6 enriched in patients with persistent disease. Stromal o Fibroblasts expressed inflammatory ligands including THY1, CXCL1, CXCL6 and OSMR
Lafzi 2024 (17)	Spatial transcriptomics *(10x Genomics Visium)*	UC: 2 HC: 4	Pathogenesis	Epithelial o Increased M-cell populations during inflammation. Cell–cell interactions o Ligand–receptor interactions between epithelial cells and fibroblasts included ADAM15–ITGA5 and VEGFA–PDGFRA signalling. Complement signalling o Differential co-occurrence of C3 with inflammatory mediators including MDK and SAA1.
Lyu 2022 (69)	Spatial transcriptomics and proteomics *(Nanostring GeoMx DSP)*	UC: 42 HC: 36	Pathogenesis	Enterochromaffin cells o Increased expression of genes involved in mucosal regeneration including REG1A, OLFM4 and CD74.
vanUnen 2022 (52)	Spatial proteomics *(Fluidigm Hyperion)*	UC: 4 HC: 4	Pathogenesis	Spatial organisation o Cellular aggregates composed of CD11c+ myeloid cells, CD66b+ neutrophils and CD4+ T cells located beneath the epithelial layer in UC.
Venkat 2025 (53)	Spatial transcriptomics and proteomics *(Nanostring GeoMx DSP*)	UC: 45 HC: 0	Pathogenesis	Fibroblasts o Increased intestinal inflammatory activated fibroblasts (IIAFs) in severe UC. o IIAFs expressed genes associated with fibrosis and remodelling including COL7A1, TGFB3, IL11, WNT2 and TWIST1.
Zhang 2024* (59)	Spatial transcriptomics and proteomics *Nanostring GeoMx DSP)*	UC: 4 HC: 2	Pathogenesis	Lipid metabolism o Increased SMPD1 expression in both immune and non-immune compartments. o Sphingolipid signalling o Reduced CERS2 and CERS6, indicating altered sphingolipid metabolism in UC.
Holman 2025* (55)	Spatial proteomics *(Akoya Biosciences CODEX)*	UC: 29 (on TNFi) HC: 5	Treatment response	TNFi treatment o Adaptive immune niches o Reduction of lymphoid aggregates and adaptive immune cellular neighbourhoods. o Innate immune niches o Persistent neutrophil-enriched niches expressing TNFR2 and TNFRSF9, suggesting ongoing inflammation despite therapy suggestive of treatment resistance
Kim 2024* (56)	Spatial transcriptomics and proteomics *(Digital Spatial* Profiling)	UC: 54 (54 on TNFi, 29 responders, 25 non-responders) HC: 2	Treatment response	TNFi response o Responders: Higher expression of colonocyte maturation genes including BEST4, CA2, CA7 and PHGR1. o Non-responders: Reduced epithelial maturation signatures and increased crypt distortion and atrophy.
Mayer 2023 (54)	Spatial proteomics *(Akoya Biosciences CODEX)*	UC:29 (15 on TNFi, 6 responders, 7 non-responders) HC:5	Pathogenesis; Treatment response	Immune architecture o UC tissue contained diverse immune neighbourhoods including lymphoid aggregates and mixed immune niches. Disease severity o Increased expression of TNFRSF9 correlated with disease activity. Treatment effects o TNFi therapy reduced adaptive immune cell interactions but granulocyte-rich niches persisted.

*abstract only, scRNA-seq, Single-cell RNA sequencing; UC, Ulcerative colitis; HC, Healthy control; Treg, Regulatory T cell; TRM, Tissue-resident memory T cell; IEL, intra-epithelial lymphocytes; TNFi, Tumour necrosis factor inhibitor.

↓, reduced; ↑, increased

### 3.1 Pathogenesis of UC from single-cell RNA sequencing studies

#### 3.1.1 Innate immune cells

Twelve studies reported on the gene expression profiles of innate immune cells, consistently implicating myeloid cell activation and pathways involved in tissue damage, repair, and fibrosis in the pathogenesis of UC.

The largest studies, by Smillie et al. and Thomas et al., identified an expansion of myeloid cells, inflammatory monocytes, and mast cells in UC tissue, each characterised by distinct transcriptional signatures (22, 23). Myeloid cells showed upregulation of *NRG1* and *RETN*, genes associated with epithelial repair and pro-inflammatory signalling, respectively. Inflammatory monocytes expressed *S100A8*, a key mediator of inflammation, as well as Oncostatin M (OSM), a cytokine linked to chronic inflammation and tissue remodelling. Notably, OSM was also found to be upregulated in dendritic cells (22, 23).

Zhou et al. identified a distinct population of inflammation-associated macrophages characterised by *SPP1* expression, along with *IL7R*, multiple matrix metalloproteinases, and the neutrophil-attracting chemokines *CXCL5* and *CXCL1*. This transcriptional profile suggests a role in extracellular matrix remodelling and in promoting neutrophil recruitment and myeloid immune activation (24). Similar findings were reported by Jha et al. and Mitsialis et al., who also observed an expansion of myeloid cells expressing a broad set of pro-inflammatory genes, including *IL1B, CD14, FCGR3A/B, S100A8/A9, FPR1, FPR2, CLEC4A, TREM1, CXCR1/2*, and *CXCL1*. This gene signature signifies ongoing innate immune activation and signal amplification, consistent with epithelial damage and microbial translocation, driving chemotaxis and activation of monocytes and macrophages, and highlighting neutrophil-mediated inflammatory processes in UC pathogenesis (25, 26).

Consistent with these transcriptional findings, epithelial damage and ulceration, together with neutrophilic infiltration of the lamina propria, constitute core histopathological features of UC and form key components of validated histological activity indices, including the Nancy Index, Robarts Histopathology Index, and Geboes score (27–29).

Mennillo et al. reported upregulation of *TIMP1, TYMP*, and *SOD2* in mononuclear phagocytes, suggesting that these innate immune cells may contribute not only to host defence and antioxidant protection (*SOD2*), but also to tissue repair through inhibition of extracellular matrix degradation with a potential trade-off of fibrosis (*TIMP1*), and to angiogenesis and remodelling within chronically inflamed tissue (*TYMP*) (3).

#### 3.1.2 Adaptive immune cells

The healthy colonic mucosa is enriched in IgA^+^ plasma cells, which preserve microbial balance and protect the epithelium by preventing commensal and pathogenic bacteria from adhering or translocating, a process achieved through non-inflammatory immune exclusion. However, six studies reported a shift from IgA^+^ to IgG^+^ plasma cells in inflamed UC tissue (22, 23, 25, 30–32). This suggests a loss of tolerance and a shift toward pro-inflammatory antibody responses. Among these, Scheid et al., focused primarily on plasma cells and found that IgG^+^ plasma cells overexpressed *XBP1, DERL1, ERLEC1*, and *TMBIM6*, genes indicative of sustained endoplasmic reticulum (ER) stress and activation of adaptive pathways such as the unfolded protein response (UPR) and ER-associated degradation (ERAD). This profile reflects metabolic and stress adaptations characteristic of a high secretory phenotype, which may in turn exacerbate mucosal inflammation (30). Although not observed in larger studies, three smaller papers reported an expansion of *CXCR3+* plasmoblasts and *CXCR3+CCR4+* naïve B cells in UC tissue, indicating potential activation and trafficking of B cell subsets in the inflamed colon (25, 32, 33).

Despite these tissue-level observations, clinical translation has been limited. A randomized, placebo-controlled trial of the anti-CD20 B-cell–depleting antibody rituximab failed to demonstrate efficacy in active ulcerative colitis, likely reflecting its inability to deplete colon-resident plasma cells, which lack CD20 expression (34). In contrast, a recent single-patient report of CD19-directed CAR-T cell therapy described marked clinical and mucosal improvement in refractory UC; while uncontrolled, this observation suggests that therapeutic strategies targeting B-cell lineages may yet warrant further investigation in UC (35).

Regulatory T cells (Tregs) were found to be increased in inflamed UC tissue across five studies and displayed a pro-inflammatory gene signature (3, 22, 25, 30, 36). While this expansion has been interpreted as a compensatory attempt to control mucosal inflammation, several studies noted altered transcriptional profiles suggesting impaired suppressive function or even a shift toward a pathogenic phenotype (22, 25, 30). In line with this, Smillie et al. identified Tregs as one of the principal cellular sources of TNF in UC, highlighting the concept of Treg plasticity whereby chronic inflammation drives Tregs toward a pro-inflammatory, Th1-like state. Such findings help explain the paradox of increased Treg abundance yet uncontrolled inflammation and suggest that Treg dysfunction may actively contribute to UC pathogenesis (22). TNF has also been found to be overexpressed in memory T cells and effector memory T cells (23, 37). An early clinical report from a Phase 1, dose−escalation trial of adoptive Treg transfer in 8 refractory UC patients showed promise with clinical response in two-thirds and fall in faecal calprotectin at 12 weeks (38). Potentially in the future, CAR-Treg therapies may be designed to target this suppressive axis (39).

Multiple studies reported on CD8^+^ and CD4^+^ T cell populations. CD8^+^ T cells are expanded in UC tissue (22, 37). Corridoni et al. reported that CD8^+^ intraepithelial lymphocytes (IELs) and CD8^+^ tissue resident memory (T_RM_) cells over-expressed *TNFRSF9* and *CTLA4* - indicating both chronic activation of CD8^+^ T cells and the counter-regulatory attempts to limit excessive immune activation, respectively (40). Parikh et al. similarly reported upregulation of *TNFRSF9* in CD8^+^ IELs, along with *IL17R* (41).* A* subset of IL26^+^CD8^+^ T cells in Corridoni et al. expressed *CTLA4, PDCD1, TOX* and *HAVCR2* with authors suggesting that T cell exhaustion may be playing a role in the pathogenesis of UC (40, 41). Taken together, these data suggest that CD8^+^ T cells may simultaneously contribute to epithelial immune surveillance, pro-inflammatory cytokine production, and tissue damage, while exhaustion pathways attempt, but fail, to fully restrain inflammation in UC. To date, broad T-cell–targeted therapies have not been successful in UC (42). While numerous agents targeting T-cell co-stimulatory or inhibitory pathways (e.g., PD−1, CTLA−4) have been developed in oncology, agonist approaches using these targets may show promise in rheumatoid arthritis but to date, disappoint in UC (43).

In the CD4^+^ T cell compartment, Smillie et al. and Thomas et al. found overexpression of *IL17A, IL21*, and *IL21R*, indicative of pathogenic T helper (Th17 or Tfh-like) subsets which drive neutrophil recruitment, contribute to mucosal inflammation, B cell activation and IgG class switching (22, 23). Li et al. further showed that effector memory CD4+ T cells overexpressed *CCR6, CD161, IFNG* and *IL17A*, markers associated with Th17 and Th1-like inflammatory profiles (37). These data suggest that CD4^+^ T cells in UC are skewed toward pro-inflammatory effector states that promote neutrophil recruitment, tissue damage, and B cell activation, potentially driving IgG class switching. CCR6-mediated mucosal trafficking of these effector memory T cells likely sustains chronic inflammation, while co-expression of IFN-gamma and IL17A points to T cell plasticity (Th1/Th17 hybrid or plastic T cells) and the generation of hybrid pathogenic phenotypes contributing to ongoing epithelial injury (44).

An expansion of CXCL13^+^ T peripheral helper (T_PH_) cells was also observed in UC tissue. These cells overexpressed genes involved in immune checkpoint regulation and T cell exhaustion, including *CTLA4, PDCD1, TIGIT*, and *CD200* (29). Similarly, Th17 cells were found to overexpress multiple immune checkpoint molecules, such as *LAG3, CTLA4, TNFRSF4, TNFRSF18*, and *HAVCR2*, across several studies, highlighting the interplay between chronic inflammation and T cell regulatory pathways in UC (23, 32, 37).

#### 3.1.3 Stromal cells

Several studies identified the expansion of fibroblasts, endothelial cells, and pericytes in UC tissue (3, 22, 23, 26, 37, 45–50). Gene expression profiles suggest that activated fibroblasts play a key role in driving inflammation and fibrosis.

Kinchen et al. first described a distinct fibroblastic-reticular cell population expanded in UC tissue, characterized by the expression of *CCL19, CCL21, TNFSF14 (LIGHT), IL33*, and *MHC II*. This gene expression profile suggests a role in immune cell recruitment, fibroblast-immune interactions, and activation of the NF-κB pathway (46).

Smillie et al. and Thomas et al. supported these findings, reporting that fibroblasts from inflamed UC tissue upregulated genes associated with fibroblast activation, tissue remodelling, and fibrosis, including *FAP, WNT2*, and several neutrophil-recruiting chemokines (CXCL3, CXCL5, CXCL6, CXCL8) and their receptors (*CXCR1, CXCR2*). These transcriptional profiles further emphasise the role of fibroblasts in shaping the pro-inflammatory tissue microenvironment in UC (22, 23).

Additional characterisation of fibroblast subtypes was provided by Friedrich et al., who focused specifically on the stromal compartment of IBD tissue in their scRNA-seq study. This study identified a distinct cluster of inflammatory associated fibroblasts (IAFs) that expressed particularly high levels of *CXCL1, CXCL2, CXCL3, CXCL5, CXCL6*, and *CXCL8*, chemokines known to drive neutrophil recruitment. The *CXCR1* and *CXCR2* ligands were significantly upregulated on IAFs compared to other stromal subsets, indicating that this fibroblast population may play a central role in neutrophil infiltration and the maintenance of mucosal inflammation (50). Consistent with these findings, Zhou et al. also identified a CHI3L1^+^ fibroblast population enriched in inflamed UC tissue. These fibroblasts expressed high levels of *CHI3L1*, multiple matrix metalloproteinases (*MMP1, MMP3, MMP10)*, and neutrophil-attracting chemokines (*CXCL1, CXCL2, CXCL3, CXCL8*). This transcriptional overlap with the SPP1^+^ macrophage population described in the same study suggests coordinated fibroblast–macrophage crosstalk within inflamed UC tissue (24).

Oliver et al. created an integrative atlas of scRNA-seq studies in IBD, revealing that inflammatory fibroblasts in IBD patients exhibit transcriptional profiles more closely resembling oral mucosa fibroblasts than those of homeostatic intestinal fibroblasts (19).

In UC, across 3 studies, endothelial cells exhibited increased expression of genes associated with inflammation (*LCN2, JUN, MHC*), immune regulation (*CD59, S100A6*), and vascular remodelling (*PDGFB, TM4SF1, MGP*) (3, 37, 48). Upregulation of *TIMP1, PRKCDBP*, and *TPM4* suggests active extracellular matrix remodelling and endothelial-to-mesenchymal transition, while reduced *TXNIP* and *FABP5* expression indicates altered oxidative stress and metabolic processes (3, 37).

#### 3.1.4 Epithelial cells

Significant alterations in epithelial cell populations were observed across the included studies (22, 23, 37, 41, 49).

Smillie et al. identified a novel BEST4/OTOP2^+^ absorptive colonocyte population, marked by elevated expression of ITLN1 and IL1R2 and reduced expression of metallothionein genes. This population was observed in both healthy individuals and those with ulcerative colitis (UC), a finding corroborated by Parikh et al. (22, 41). In the same study, Parikh et al. described a crypt-bottom phenotype in UC, where genes normally restricted to the crypt base, *OLFM4, SPINK1*, and *SPINK4*, were aberrantly expressed throughout the crypt axis (41). These changes may suggest a positive feedback loop driven by increased epithelial cell turnover in inflamed tissue. Additionally, goblet progenitor cells were reduced in inflamed regions, indicating impaired epithelial differentiation (37, 41).

The integrated scRNA-seq atlas by Oliver et al. revealed Paneth cell metaplasia in both inflamed and macroscopically non-inflamed colon in CD and UC patients. This indicates that chronic, long-standing epithelial injury in UC can drive a stem cell fate switch toward a Paneth-like lineage, producing cells with both barrier-repair and immune-recruiting potential. It also underscores a strength of this integrative study design: rare cell populations that may be obscured within clusters in individual studies can be uncovered through combined analysis (19).

Changes in immune-sensing epithelial populations were also evident. *TRPM5*^+^ tuft cells, were decreased in inflamed tissue, and expressed genes related to signal transduction and lipid metabolism (e.g., *AZGP1, BMX, ALOX5, PTGS1, IL17RB*) (37). Notably, M cells, responsible for antigen sampling, were expanded in UC and expressed *NR5A2*, *CCL20*, and *JAK2*, indicating enhanced immune surveillance (22).

### 3.2 Treatment response in UC from single-cell RNA sequencing studies

Single-cell RNA sequencing (scRNA-seq) studies have identified key cellular and molecular signatures associated with treatment non-response in UC.

Thomas et al. reported that non-response to anti-TNF therapy was associated with heightened activation of myeloid cells and inflammatory monocytes, higher numbers of γδ T cells, and decreased expression of *MUC2* and *MUC5* in goblet cells (16).

A consistent finding across multiple studies was the association between inflammatory-associated fibroblasts (IAFs) and treatment resistance. Thomas et al. found that fibroblasts expressing *THY1, CXCL1, CXCL6, PDPN*, and *OSMR* were enriched in non-responders to adalimumab, suggesting that fibroblast-driven inflammation may contribute to anti-TNF resistance (16). All samples in this study were obtained from biologic-naïve patients with moderate to severe ulcerative colitis, however, disease duration at the time of biopsy varied among participants.

These results were supported by Smillie et al., who identified IAFs overexpressing *IL13RA2, TNFRSF11B*, and *IL11* in anti-TNF non-responders. They further highlighted the role of the Oncostatin M (OSM) signalling pathway, with OSM being produced by inflammatory monocytes and dendritic cells, and its receptor *OSMR* most enriched in IAFs. They proposed that the expansion of these cell types and their interaction via the OSM–OSMR axis may underlie treatment resistance (22).

Similar patterns were observed by Hsu et al. in the context of vedolizumab non-response, where overexpression of *IL1A, IL1RN*, OSM, and *CCL20* in macrophages and inflammatory monocytes, as well as OSM in dendritic cells, was associated with treatment failure (51).

Friedrich et al. provided further evidence of the role of IAFs in treatment resistance. They identified an *IL-1R*–driven inflammatory fibroblast–neutrophil recruitment pathway, characteristic of the M4/M5-high pathotype. This pathotype, marked by deep ulceration, neutrophilic infiltration, and fibroblast activation, was strongly associated with nonresponse to multiple therapies including steroids, anti-TNFs, and anti-integrins, highlighting *IL-1R* signalling as a potential therapeutic target in treatment-refractory UC (50).

Mennillo et al. reported that treatment with vedolizumab was associated with a general reduction in activated fibroblasts, monocytes, macrophages, and mast cells compared to UC patients on mesalazine treatment. Notably, the study reported a significant decrease in myeloid dendritic cells. Interestingly, despite vedolizumab’s presumed impact on lymphocyte trafficking, no significant changes were observed in lymphocyte subsets. In contrast, epithelial cell numbers increased in treated patients, accompanied by a reduction in deep crypt secretory cells, suggesting a shift toward mucosal healing (3).

## 4 Key biological insights relevant to pathogenesis and treatment responses of UC from ST and SP studies

### 4.1 Resolve the cellular composition of the UC microenvironment and map its spatial organization

Nine of the included studies focus on characterizing the inflammatory microenvironment in UC, highlighting key cellular neighbourhoods and interactions that define inflamed mucosal tissue.

A consistent finding across studies is the increased proximity between various immune cell subsets and epithelial cells. VanUnen et al. used spatial proteomic techniques to demonstrate the co-localization of immune cell aggregates, comprised of CD4+ T cells, neutrophils and several antigen presenting cells, just below the epithelial layer (52). Jha et al. reported that the UC microenvironment was characterised by loss of mature epithelial cells, and an influx of immune cells with enhanced proximity between absorptive epithelial cells and neutrophils, suggesting a key role for neutrophils in driving epithelial damage and inflammation (26). This finding is supported by Garrido-Trigo et al. who found that neutrophils were distributed throughout the lamina propria but were most densely localized in crypt abscesses and ulcerated areas, highlighting their contribution to tissue injury and disease progression (49).

In the study by Du et al., tissue-resident macrophages were replaced by infiltrating macrophages, leading to changes in local cellular neighbourhoods. This shift resulted in TNFα production moving from epithelial regions to inflammatory lymphocytes in the lamina propria, driven by *IL-1β* produced by these infiltrating macrophages (36). Garrido-Trigo et al. identified a distinct multicellular hub within the inflamed UC microenvironment, composed of neutrophils, macrophages, inflammatory fibroblasts, and inflammatory monocytes. Inflammation-dependent macrophages showed strong spatial association with inflammatory fibroblasts, which expressed factors such as CSF2, CSF3, and prostaglandin-producing enzymes, known drivers of macrophage activation and differentiation. These findings suggest substantial crosstalk between the two cell types (49).

Venkat et al. reported an intestinal inflammatory activated fibroblast (IIAF) population correlated with histological disease severity, characterised by expression of pro-fibrotic and remodelling genes including *COL7A1*, *TGFB3*, *IL11*, *WNT2*/*5A, TWIST1/2*, and *COL21A1 (*53*).*

Mayer et al. used spatial proteomic techniques to demonstrate how cellular neighbourhoods evolve with disease severity in UC. They found that increases in granulocyte-rich, mixed immune, and lamina propria neighbourhoods were associated with higher Mayo scores, while luminal and basal epithelial neighbourhoods decreased as disease severity worsened. Additionally, they showed that the functional state of individual cells is shaped by their spatial context. For example, the frequency of TNFR2-expressing neutrophils varied significantly depending on their location, with the highest expression observed in inflamed vasculature neighbourhoods, compared to inflamed stroma or mixed immune regions (54).

### 4.2 Integrate cellular composition analysis with spatial co-localization to better distinguish inflammatory microenvironments across drug response subtypes

In relation to treatment response, five studies looked at response to anti-TNF therapy (specifically infliximab (25, 54–56) and adalimumab (23)) and one study looked at response to vedolizumab (3).

Mayer et al. compared anti-TNF–treated patients with disease severity–matched UC controls and identified treatment-associated changes in tissue architecture. Anti-TNF therapy was linked to reduced T cell frequencies, increased epithelial cell abundance, and a decrease in lymphoid aggregates and B cell follicles. In contrast, innate immune populations, such as granulocytes, remained largely unaffected, suggesting that these innate niches may be resistant to therapy, while adaptive immune neighbourhoods tend to normalise with treatment. However, the ability to predict treatment response based on spatial features proved variable and inconsistent, despite testing both handcrafted metrics (cell ratios, cell–cell interactions, neighbourhood composition) and deep learning–derived image features using convolutional neural networks applied to Voronoi tissue patches. The authors concluded that although spatial signals exist, they appeared ‘to be lost amidst the complex resistance-associated patient heterogeneities’ (54). Beyond differences in tissue architecture and immune organisation, demographic and clinical factors such as sex, disease duration and severity, and age compounded variability and hindered reliable prediction of response.

An abstract by Holman et al. similarly reported that anti-TNF therapy led to the normalization of adaptive immune cellular neighbourhoods, while innate immune niches, particularly neutrophil-enriched granulocyte niches, persisted. These neutrophil-dominated neighbourhoods continued to express pro-inflammatory markers such as TNFR2 and CD137, indicating ongoing mucosal inflammation which may be responsible for resistance to therapy (55).

Interestingly both papers, reported higher anti-TNF responsiveness in females, compared to males, which was related to enrichment of the adaptive immune components in female patients (54, 55).

Kim et al. supported Mayer et al.’s findings, that epithelial neighbourhoods expanded following anti-TNF treatment. Their abstract also noted that crypt distortion and atrophy were more pronounced in non-responders, suggesting impaired epithelial repair may contribute to treatment failure (56).

Similarly, in the context of vedolizumab therapy, a more robust epithelial crypt base and lower levels of innate immune cells were associated with treatment response. Additionally, in Mennillo et al. non-response was linked to a higher abundance and closer proximity of activated fibroblasts and mononuclear phagocytes (MNPs) (3).

### 4.3 Utilise spatially defined ligand-receptor interactions as potential biomarkers for predicting drug response and identify novel therapeutic targets

Lafzi et al. applied a computational framework to spatial transcriptomic data to identify spatially co-occurring ligand receptor (LR) pairs, focusing on interactions arising from spatial proximity rather than differential gene expression. Through this approach, they identified co-occurring LR pairs enriched in inflammatory cellular neighbourhoods, including *ADAM15*–*ITGA5* and *VEGFA*–*PDGFRA*, linking M cells and fibroblasts. Additional interactions, such as *CXCL5*–*ITGAM* in neutrophil-rich regions and *SECTM1*–*CD7* within the crypt epithelium, underscore the importance of spatially restricted immune–epithelial crosstalk (17). *SECTM1* stimulates T cell proliferation and monocyte migration and its expression has been shown to differ between paediatric responders and non-responders to anti-TNF therapy, supporting its potential as a biomarker for predicting treatment response (57).

The identification of *IL-1β*, predominantly produced by macrophages, as a key upstream driver of TNFα production by T and B cells highlights a potentially important ligand receptor interaction within the UC inflammatory microenvironment. Although targeting *IL-1β*, or the specific macrophage population responsible for its production, could offer a promising therapeutic strategy, a phase II multi-centre, randomised, placebo-controlled trial of the *IL-1* receptor antagonist anakinra failed to demonstrate efficacy in acute severe ulcerative colitis (36, 58).

Thomas et al. identified resistance to anti-TNF treatment as being characterised by specific signalling pathways, including the myeloid–vascular *CXCL10*–*ACKR1* axis, fibroblast-derived ligands (*THY1, CXCL1, CXCL6, CCL19*), *IL-21*–*IL-21R* signalling, and widespread interferon (IFN) responses. These findings highlight IFN-driven signalling as a promising target for therapeutic intervention. The authors also support the rationale for using JAK inhibitors and IL-23p19 antagonists, which modulate IFN-related pathways, as potential strategies in non-responders to anti-TNF therapy (23).

## 5 Risk of bias

Of the 29 included studies, 23 had a low risk of bias, while 6 had a moderate risk. Three abstracts were included; however, due to limited reporting on participant characteristics and methodology, an overall risk of bias assessment was not assigned to these studies (55, 56, 59).

Study methods were well-documented, outcomes clearly defined, and bioinformatics pipelines transparently reported. However, 21 studies did not account for key biological confounders, such as disease severity, disease duration and treatment history, or failed to provide relevant details. This introduces risk of overgeneralisation, as findings may not be representative of the whole UC population, and reduced reliability, as observed effects could be influenced by unmeasured variables. Fifteen studies did not specify whether samples were taken from the same colonic location or whether healthy control samples were appropriately matched, limiting comparability between studies.

Among the 8 studies investigating treatment response, only 3 clearly defined and consistently applied criteria for treatment failure, such as the STRIDE II guidelines (60).

**Table 3** summarizes the risk of bias assessment for all included studies.

**Table 3:** Risk of bias assessment for each study – a clinical perspective.

Study ID	Confounding		Classification of disease			Selection of participants/Cells		Deviation from protocol	Missing data	Measurement of outcomes		Selection of reported results		Overall risk of bias
	Q1	Q2	Q3	Q4	Q5	Q6	Q7	Q8	Q9	Q10	Q11	Q12	Q13	
Boland 2020	PN	PY	PY	N/A	N/A	Y	Y	Y	Y	Y	Y	Y	PY	Low
Chen 2021	PN	PY	PY	N/A	N/A	PY	PY	Y	Y	Y	Y	PY	Y	Low
Corridoni 2020	NI	Y	NI	N/A	N/A	Y	Y	Y	Y	Y	Y	PY	Y	Low
Kinchen 2018	PY	PY	NI	N/A	N/A	Y	PY	Y	Y	Y	Y	Y	Y	Low
Korsunsky 2022	N	PY	PN	N/A	N/A	PY	Y	Y	Y	Y	Y	PY	Y	Low
Li 2021	PY	PY	Y	N/A	N/A	Y	PY	Y	Y	N	Y	PY	Y	Low
Luo 2022	PN	PY	NI	N/A	N/A	Y	PY	Y	Y	Y	PY	PN	Y	Moderate
Mitsialis 2020	NI	Y	NI	N/A	N/A	Y	PY	Y	Y	Y	Y	PY	Y	Low
Mo 2021	NI	Y	PY	N/A	N/A	Y	Y	Y	Y	Y	Y	PN	Y	Low
Parikh 2019	PY	Y	NI	N/A	N/A	Y	PY	Y	PY	Y	Y	PY	Y	Low
Scheid 2023	PN	Y	NI	N/A	N/A	Y	PY	Y	PY	Y	Y	PY	Y	Low
Smillie 2019	PN	Y	PN	Y	N/A	Y	PY	Y	PY	Y	Y	Y	Y	Low
Uzzan 2022	PN	Y	NI	N/A	N/A	PY	PY	PY	NI	Y	PY	PY	Y	Low
Zhou 2025	PN	Y	NI	N/A	N/A	Y	Y	Y	Y	Y	Y	PY	Y	Low
Friedrich 2021	Y	Y	NI	N/A	NI	PY	Y	Y	PY	Y	Y	PY	Y	Low
Hsu 2023	PN	Y	NI	N/A	Y	PY	Y	Y	PY	Y	Y	PY	Y	Low
Du 2023	PN	NI	PN	N/A	PY	Y	PY	PY	NI	Y	PY	PN	PY	Moderate
Garrido-Trigo 2023	PN	Y	NI	Y	N/A	Y	Y	Y	Y	Y	Y	PY	Y	Low
Jha 2023	PN	Y	NI	N/A	PN	Y	Y	Y	Y	Y	Y	PY	Y	Moderate
Mennillo 2024	PN	PY	PY	N/A	PN	Y	Y	Y	Y	Y	Y	PY	Y	Low
Thomas 2024	PY	Y	PY	N/A	Y	Y	Y	Y	Y	Y	Y	PY	Y	Low
Lafzi 2024	NI	PY	NI	N/A	N/A	PY	NI	Y	PY	PY	Y	PN	Y	Moderate
Lyu 2022	PY	Y	PY	N/A	N/A	Y	NI	Y	Y	Y	Y	PY	Y	Low
vanUnen 2022	PY	Y	PY	Y	N/A	Y	PY	Y	PY	Y	Y	PY	Y	Low
Venkat 2025	PN	NI	NI	N/A	N/A	NI	PY	PY	NI	N	Y	PN	N	Moderate
Zhang 2024*	NI	NI	NI	Y	N/A	Y	NI	PY	NI	PN	Y	PY	NI	Abstract only
Holman 2025*	NI	NI	NI	N/A	NI	PY	NI	PY	NI	PN	Y	PY	NI	Abstract only
Kim 2024*	NI	NI	NI	N/A	PN	PY	NI	PY	NI	PN	Y	PY	NI	Abstract only
Mayer 2023	PY	NI	NI	N/A	NI	PY	NI	PY	NI	PY	Y	PY	Y	Moderate

Confounding: Q1. Did the study account for biological confounders i.e. disease severity, patient age, treatment history? Q2. Did the study account for technical confounders i.e. batch effects, differences in sample collection and preparation? Classification of Disease: Q3. Were UC samples consistently taken from the same location? Were healthy control samples matched for location? Q4. When studies included ‘IBD’ patients, were Crohn’s disease and UC reported separately? Q5. For studies with a focus on treatment response, was treatment clearly defined and consistently applied (e.g. allowing 6 months of treatment, before defining treatment failure as per STRIDE II)? Selection of participants / cells: Q6. Were cells representative of the colonic mucosa (e.g. epithelial, stromal, immune cells)? Q7. Is cell capture, dropout events and cell viability reported and appropriately handled? Deviation from Intended Protocol: Q8. Was the experimental protocol appropriately reported? Were any deviations documented and accounted for (e.g. using batch correction methods)? Missing Data: Q9. Did the study report proportions of cells or regions excluded due to quality control? Were these handled appropriately? Measurement of Outcomes: Q10. Was there any experimental validation of key findings (e.g. via qPCR or spatial validation with *in-situ* hybridisation or immunofluorescence)? Q11. Was there consistency in defining outcomes (e.g. differential expression thresholds, clustering methods)? Selection of Reported Results: Q12. Did the study report all differentially expressed genes? Were spatially distinct regions or cellular neighbourhoods equally considered when reporting? Q13. Was there transparency in bioinformatics pipelines?

Responses Yes (Y), Probably Yes (PY), Probably No (PN), No (N) and No Information (NI).

*abstract only.

## 6 Discussion, clinical perspectives and recommendations

This systematic review synthesises recent advances in our understanding of ulcerative colitis (UC) pathogenesis and treatment response, drawing on insights from single-cell and spatial transcriptomic and proteomic studies.

Single-cell RNA sequencing (scRNA-seq) has transformed the field by enabling high-resolution profiling of immune, stromal, and epithelial cell populations in the UC colonic mucosa (22–26, 30–33, 36, 37, 40, 41, 45–50). However, scRNA-seq techniques have important limitations. In many studies, researchers pre-select specific cell populations for sequencing (typically using sorting techniques such as FACS) to enrich for cells of interest and enhance transcriptional resolution. While useful for targeted analyses, this approach limits the ability to assess the relative abundance of different cell types and detect compositional shifts in response to disease or treatment. In addition, certain cell types, such as eosinophils and neutrophils, are often underrepresented due to their fragility and sensitivity to microfluidic handling (61). Rare cell types, like Paneth cells, may also be hidden within larger clusters, making it difficult to reliably capture their transcriptomes (19). Emerging technologies, such as the BD Rhapsody system, can help mitigate some of these challenges by minimising cell stress and improving cell capture efficiency (62, 63).

While scRNA-seq provides valuable insights into transcriptional states and cell-type heterogeneity, spatial technologies build upon these insights illuminating how cells are organised within the UC inflammatory microenvironment. This adds a critical layer of context, highlighting that cell function and treatment response are often governed, not only by intrinsic transcriptional states, but also by their spatial context within the tissue. However, accurate interpretation of spatial gene expression patterns often depends on high-quality single-cell reference data (64).

Despite the promise of these technologies, clinical heterogeneity remains a major limitation to interpreting and generalising findings. Many included studies were constrained by small sample sizes, inconsistent disease stratification, and limited clinical metadata. The increasing availability of large-scale single-cell datasets, and the evolution of high-throughput spatial platforms, will soon allow for the analysis of hundreds or thousands of samples. To ensure technologies yield clinically relevant insights, future research must prioritise robust study design, detailed clinical phenotyping, and standardised sampling protocols.

The Human Gut Cell Atlas recognised the growing use of scRNA-seq technologies in advancing our understanding of the gastrointestinal tract at the cellular level. In response, it highlighted key challenges and called for a more structured and coordinated approach moving forward. Its published roadmap outlines recommendations for future studies, discusses core methodologies, and provides a template for metadata collection (65). While the template includes fields related to the anatomical location of sample collection, it lacks detail and specificity on disease reporting and important clinical characteristics.

We propose that precise disease stratification and clinical phenotyping are essential when designing study cohorts. In IBD, clinical characteristics such as disease distribution, time since diagnosis, disease severity, and current or past treatments are important potential confounders. These should be carefully considered during study design, alongside the application of rigorous scientific methodology.

In this systematic review, we deliberately adopted different aims and structure when reviewing spatial and single-cell transcriptomics in UC, in part to avoid redundancy with existing and overlapping single-cell literature, and more importantly, to highlight the unique contributions of spatial approaches. By preserving tissue architecture, spatial technologies enable interrogation of cell-cell interactions, niche-specific expression profiles, and spatially restricted pathological processes, dimensions that single-cell technologies, by their dissociative nature, cannot fully capture. Our review structure was therefore tailored to emphasise these spatially resolved insights, allowing us to more clearly define the added value of spatial approaches in UC.

We propose three key aims to guide future studies. First, to more precisely define the UC inflammatory microenvironment, across clearly defined patient subsets. While current spatial studies have begun to map the cellular composition and organisation of inflamed mucosa, they have yet to drill down into the spatial heterogeneity of different UC phenotypes. Future work could compare spatial signatures across disease subtypes and stages to identify shared and divergent features.

Second, spatial technologies should be applied to better understand treatment response. Initial findings suggest that certain spatial features, such as the persistence of innate immune niches or loss of epithelial integrity, may correlate with non-response to treatment. However, these features have not yet shown consistent predictive value (54). Larger, stratified studies with temporal sampling before and after treatment, are required to determine whether spatial organisation can be used prospectively to guide therapeutic decisions.

Third, the analysis of spatially restricted ligand–receptor interactions offers an opportunity to uncover novel biomarkers and therapeutic targets. A small number of studies have identified candidate pathways, such as IL-1β-driven macrophage–lymphocyte signalling or fibroblast–endothelial crosstalk, but this area remains in its infancy (17, 50). To date, no spatially defined interactions have translated into clinically actionable biomarkers or therapies.

The large and complex datasets produced from spatial transcriptomic technologies has led to the rapid development of innovative machine learning tools, primarily based on deep learning techniques. Integrating spatial transcriptomic data with deep learning applied to widely available digitalised haematoxylin and eosin (H&E) slides holds promise as a more scalable, cost-effective and clinically translatable approach (66, 67). For example, the recently published tumour immune microenvironment spatial (TIMES) score leverages spatial expression patterns to predict hepatocellular carcinoma recurrence from a single uploaded H&E slide. It outperforms existing risk stratification tools and illustrates the potential for this technology to be translated into clinical practice (68).

In conclusion, spatial and single-cell technologies are transforming how we study UC. By focusing future spatial research around the aims outlined above, and through close collaboration, with careful attention to cohort design and sample stratification, we can move toward a mechanistically precise, spatially resolved framework for understanding and treating UC.

## Data Availability

The original contributions presented in the study are included in the article/**Supplementary Material**. Further inquiries can be directed to the corresponding author.

## References

[1] Lin D Jin Y Shao X Xu Y Ma G Jiang Y . Global, regional, and national burden of inflammatory bowel disease, 1990-2021: Insights from the global burden of disease 2021. Int J Colorectal Dis. (2024) 39:139. doi: 10.21203/rs.3.rs-4810674/v1. PMID: 39243331 PMC11380638

[2] Khor B Gardet A Xavier RJ . Genetics and pathogenesis of inflammatory bowel disease. Nature. (2011) 474:307–17. doi: 10.1038/nature10209. PMID: 21677747 PMC3204665

[3] Mennillo E Kim YJ Lee G Rusu I Patel RK Dorman LC . Single-cell and spatial multi-omics highlight effects of anti-integrin therapy across cellular compartments in ulcerative colitis. Nat Commun. (2024) 15:1493. doi: 10.1038/s41467-024-45665-6. PMID: 38374043 PMC10876948

[4] Saez A Herrero-Fernandez B Gomez-Bris R Sánchez-Martinez H Gonzalez-Granado JM . Pathophysiology of inflammatory bowel disease: Innate immune system. Int J Mol Sci. (2023) 24:1526. doi: 10.3390/ijms24021526. PMID: 36675038 PMC9863490

[5] Lasa JS Olivera PA Danese S Peyrin-Biroulet L . Efficacy and safety of biologics and small molecule drugs for patients with moderate-to-severe ulcerative colitis: a systematic review and network meta-analysis. Lancet Gastroenterol Hepatol. (2022) 7:161–70. doi: 10.1016/s2468-1253(21)00377-0. PMID: 34856198

[6] Moss AC . Approach to treatment failure in inflammatory bowel disease. Gastroenterol Hepatol (N Y). (2022) 18:360–3. doi: 10.1097/mog.0000000000000449. PMID: 36398141 PMC9666824

[7] Raine T Danese S . Breaking through the therapeutic ceiling: What will it take? Gastroenterology. (2022) 162:1507–11. doi: 10.1053/j.gastro.2021.09.078. PMID: 34995533

[8] Lamb CA Saifuddin A Powell N Rieder F . The future of precision medicine to predict outcomes and control tissue remodeling in inflammatory bowel disease. Gastroenterology. (2022) 162:1525–42. doi: 10.1053/j.gastro.2021.09.077. PMID: 34995532 PMC8983496

[9] Little RD Jayawardana T Koentgen S F Z Connor J Boussioutas A . Pathogenesis and precision medicine for predicting response in inflammatory bowel disease: advances and future directions. eGastroenterology. (2024) 2:e100006. doi: 10.1136/egastro-2023-100006. PMID: 39944752 PMC11770437

[10] Montero-Meléndez T Llor X García-Planella E Perretti M Suárez A . Identification of novel predictor classifiers for inflammatory bowel disease by gene expression profiling. PLoS One. (2013) 8:e76235. doi: 10.1371/journal.pone.0076235. PMID: 24155895 PMC3796518

[11] Carey R Jurickova I Ballard E Bonkowski E Han X Xu H . Activation of an IL-6:STAT3-dependent transcriptome in pediatric-onset inflammatory bowel disease. Inflammatory Bowel Dis. (2007) 14:446–57. doi: 10.1002/ibd.20342. PMID: 18069684 PMC2581837

[12] Noble CL Abbas AR Cornelius J Lees CW Ho G-T Toy K . Regional variation in gene expression in the healthy colon is dysregulated in ulcerative colitis. Gut. (2008) 57:1398–405. doi: 10.1136/gut.2008.148395. PMID: 18523026

[13] Li X Wang C-Y . From bulk, single-cell to spatial RNA sequencing. Int J Oral Sci. (2021) 13:36. doi: 10.1038/s41368-021-00146-0. PMID: 34782601 PMC8593179

[14] Gudiño V Bartolomé-Casado R Salas A . Single-cell omics in inflammatory bowel disease: recent insights and future clinical applications. Gut. (2025) 74:1335–1345. doi: 10.1136/gutjnl-2024-334165, PMID: 39904604

[15] Liu L Davidorf B Dong P Peng A Song Q He Z . Decoding the mosaic of inflammatory bowel disease: Illuminating insights with single-cell RNA technology. Comput Struct Biotechnol J. (2024) 23:2911–23. doi: 10.1016/j.csbj.2024.07.011. PMID: 39421242 PMC11485491

[16] Danan CH Katada K Parham LR Hamilton KE . Spatial transcriptomics add a new dimension to our understanding of the gut. Am J Physiol Gastrointest Liver Physiol. (2023) 324:G91–8. doi: 10.1152/ajpgi.00191.2022. PMID: 36472345 PMC9870576

[17] Lafzi A Borrelli C Baghai Sain S Bach K Kretz JA Handler K . Identifying spatial co-occurrence in healthy and inflamed tissues (ISCHIA). Mol Syst Biol. (2024) 20:98–119. doi: 10.1038/s44320-023-00006-5. PMID: 38225383 PMC10897385

[18] Sterne JA Hernán MA Reeves BC Savović J Berkman ND Viswanathan M . ROBINS-I: a tool for assessing risk of bias in non-randomised studies of interventions. Bmj. (2016) 355:i4919. doi: 10.1136/bmj.i4919. PMID: 27733354 PMC5062054

[19] Oliver AJ Huang N Bartolome-Casado R Li R Koplev S Nilsen HR . Single-cell integration reveals metaplasia in inflammatory gut diseases. Nature. (2024) 635:699–707. doi: 10.1038/s41586-024-07571-1. PMID: 39567783 PMC11578898

[20] Armingol E Officer A Harismendy O Lewis NE . Deciphering cell–cell interactions and communication from gene expression. Nat Rev Genet. (2021) 22:71–88. doi: 10.1038/s41576-020-00292-x. PMID: 33168968 PMC7649713

[21] Wang N Song Y Hong W Mo H Song Z Dai W . Spatial single-cell transcriptomic analysis in breast cancer reveals potential biomarkers for PD1 blockade therapy. (2024). doi: 10.21203/rs.3.rs-4376986/v2

[22] Smillie CS Biton M Ordovas-Montanes J Sullivan KM Burgin G Graham DB . Intra- and inter-cellular rewiring of the human colon during ulcerative colitis. Cell. (2019) 178:714–730.e22. doi: 10.1016/j.cell.2019.06.029. PMID: 31348891 PMC6662628

[23] Thomas T Friedrich M Rich-Griffin C Pohin M Agarwal D Pakpoor J . A longitudinal single-cell atlas of anti-tumour necrosis factor treatment in inflammatory bowel disease. Nat Immunol. (2024) 25:2152–65. doi: 10.1038/s41590-024-01994-8. PMID: 39438660 PMC11519010

[24] Zhou P Tang T Zhao P Wang Q Hu X Si J . Unveiling the hidden dance: SPP1 + macrophages identified in ulcerative colitis reveal crosstalk with CHI3L1 + fibroblasts. J Transl Med. (2025) 23:567. doi: 10.1186/s12967-025-06565-5, PMID: 40399882 PMC12093798

[25] Mitsialis V Wall S Liu P Ordovas-Montanes J Parmet T Vukovic M . Single-cell analyses of colon and blood reveal distinct immune cell signatures of ulcerative colitis and Crohn's disease. Gastroenterology. (2020) 159:591–608.e10. doi: 10.1053/j.gastro.2020.04.074. PMID: 32428507 PMC8166295

[26] Jha D Al-Taie Z Krek A Eshghi ST Fantou A Laurent T . Myeloid cell influx into the colonic epithelium is associated with disease severity and non-response to anti-Tumor Necrosis Factor Therapy in patients with Ulcerative Colitis. bioRxiv. (2023) 5:2023.06.02.542863. doi: 10.1101/2023.06.02.542863, PMID: 37333091 PMC10274630

[27] Marchal-Bressenot A Salleron J Boulagnon-Rombi C Bastien C Cahn V Cadiot G . Development and validation of the Nancy histological index for UC. Gut. (2017) 66:43–9. doi: 10.1136/gutjnl-2015-310187. PMID: 26464414

[28] Geboes K Riddell R Ost A Jensfelt B Persson T Löfberg R . A reproducible grading scale for histological assessment of inflammation in ulcerative colitis. Gut. (2000) 47:404–9. doi: 10.1136/gut.47.3.404. PMID: 10940279 PMC1728046

[29] Mosli MH Feagan BG Zou G Sandborn WJ D'Haens G Khanna R . Development and validation of a histological index for UC. Gut. (2017) 66:50–8. doi: 10.1136/gutjnl-2015-310393. PMID: 26475633

[30] Boland BS He Z Tsai MS Olvera JG Omilusik KD Duong HG . Heterogeneity and clonal relationships of adaptive immune cells in ulcerative colitis revealed by single-cell analyses. Sci Immunol. (2020) 5:eabb4432. doi: 10.1126/sciimmunol.abb4432. PMID: 32826341 PMC7733868

[31] Scheid JF Eraslan B Hudak A Brown EM Sergio D Delorey TM . Remodeling of colon plasma cell repertoire within ulcerative colitis patients. J Exp Med. (2023) 220:e20220538. doi: 10.1084/jem.20220538. PMID: 36752797 PMC9949229

[32] Uzzan M Martin JC Mesin L Livanos AE Castro-Dopico T Huang R . Ulcerative colitis is characterized by a plasmablast-skewed humoral response associated with disease activity. Nat Med. (2022) 28:766–79. doi: 10.1038/s41591-022-01680-y. PMID: 35190725 PMC9107072

[33] Luo Y Liu S Li H Hou J Lin W Xu Z . Mass cytometry and single-cell transcriptome analyses reveal the immune cell characteristics of ulcerative colitis. Front Mol Biosci. (2022) 9:859645. doi: 10.3389/fmolb.2022.859645. PMID: 35813827 PMC9260076

[34] Leiper K Martin K Ellis A Subramanian S Watson AJ Christmas SE . Randomised placebo-controlled trial of rituximab (anti-CD20) in active ulcerative colitis. Gut. (2011) 60:1520–6. doi: 10.1136/gut.2010.225482. PMID: 21471566

[35] Müller F Atreya R Völkl S Aigner M Kretschmann S Kharboutli S . CD19 CAR T-cell therapy in multidrug-resistant ulcerative colitis. N Engl J Med. (2025) 393:1239–41. doi: 10.1056/NEJMc2508023, PMID: 40991907

[36] Du J Zhang J Wang L Wang X Zhao Y Lu J . Selective oxidative protection leads to tissue topological changes orchestrated by macrophage during ulcerative colitis. Nat Commun. (2023) 14:3675. doi: 10.1038/s41467-023-39173-2. PMID: 37344477 PMC10284839

[37] Li G Zhang B Hao J Chu X Wiestler M Cornberg M . Identification of novel population-specific cell subsets in Chinese ulcerative colitis patients using single-cell RNA sequencing. Cell Mol Gastroenterol Hepatol. (2021) 12:99–117. doi: 10.1016/j.jcmgh.2021.01.020. PMID: 33545427 PMC8081991

[38] Voskens C Stoica D Rosenberg M Weidinger C Vitali F Zundler S . P770 Autologous regulatory T cell transfer in patients with refractory ulcerative colitis: Interim report of a phase 1, dose-escalation trial. J Crohn's Colitis. (2023) 17:i899–900. doi: 10.1093/ecco-jcc/jjac190.0900. PMID: 41835620

[39] Jin KT Chen B Liu YY Lan HU Yan JP . Monoclonal antibodies and chimeric antigen receptor (CAR) T cells in the treatment of colorectal cancer. Cancer Cell Int. (2021) 21:83. doi: 10.1186/s12935-021-01763-9. PMID: 33522929 PMC7851946

[40] Corridoni D Antanaviciute A Gupta T Fawkner-Corbett D Aulicino A Jagielowicz M . Single-cell atlas of colonic CD8(+) T cells in ulcerative colitis. Nat Med. (2020) 26:1480–90. doi: 10.1038/s41591-020-1003-4. PMID: 32747828

[41] Parikh K Antanaviciute A Fawkner-Corbett D Jagielowicz M Aulicino A Lagerholm C . Colonic epithelial cell diversity in health and inflammatory bowel disease. Nature. (2019) 567:49–55. doi: 10.1038/s41586-019-0992-y. PMID: 30814735

[42] Giuffrida P Di Sabatino A . Targeting T cells in inflammatory bowel disease. Pharmacol Res. (2020) 159:105040. doi: 10.1016/j.phrs.2020.105040. PMID: 32585338

[43] Fisher BA van de Wiel J Turner JD Liu YS Hosack T Young J . POS0094 Pharmacodynamic activity of JNJ-67484703 in rheumatoid arthritis, ulcerative colitis and Sjogren's disease (PARIS): Results of a phase II proof of biology trial. Ann Rheumatic Dis. (2025) 84:389–90. doi: 10.1016/j.ard.2025.05.482. PMID: 41836151

[44] Shale M Schiering C Powrie F . CD4+ T-cell subsets in intestinal inflammation. Immunol Rev. (2013) 252:164–82. doi: 10.1111/imr.12039. PMID: 23405904 PMC3736165

[45] Chen E Chuang LS Giri M Villaverde N Hsu NY Sabic K . Inflamed ulcerative colitis regions associated with MRGPRX2-mediated mast cell degranulation and cell activation modules, defining a new therapeutic target. Gastroenterology. (2021) 160:1709–24. doi: 10.1053/j.gastro.2020.12.076. PMID: 33421512 PMC8494017

[46] Kinchen J Chen HH Parikh K Antanaviciute A Jagielowicz M Fawkner-Corbett D . Structural remodeling of the human colonic mesenchyme in inflammatory bowel disease. Cell. (2018) 175:372–386.e17. doi: 10.1016/j.cell.2018.08.067. PMID: 30270042 PMC6176871

[47] Korsunsky I Wei K Pohin M Kim EY Barone F Major T . Cross-tissue, single-cell stromal atlas identifies shared pathological fibroblast phenotypes in four chronic inflammatory diseases. Med. (2022) 3:481–518.e14. doi: 10.1016/j.medj.2022.05.002. PMID: 35649411 PMC9271637

[48] Mo A Nagpal S Gettler K Haritunians T Giri M Haberman Y . Stratification of risk of progression to colectomy in ulcerative colitis via measured and predicted gene expression. Am J Hum Genet. (2021) 108:1765–79. doi: 10.1016/j.ajhg.2021.07.013. PMID: 34450030 PMC8456180

[49] Garrido-Trigo A Corraliza AM Veny M Dotti I Melón-Ardanaz E Rill A . Macrophage and neutrophil heterogeneity at single-cell spatial resolution in human inflammatory bowel disease. Nat Commun. (2023) 14:4506. doi: 10.1038/s41467-023-40156-6. PMID: 37495570 PMC10372067

[50] Friedrich M Pohin M Jackson MA Korsunsky I Bullers SJ Rue-Albrecht K . IL-1-driven stromal-neutrophil interactions define a subset of patients with inflammatory bowel disease that does not respond to therapies. Nat Med. (2021) 27:1970–81. doi: 10.1038/s41591-021-01520-5. PMID: 34675383 PMC8604730

[51] Hsu P Choi EJ Patel SA Wong WH Olvera JG Yao P . Responsiveness to vedolizumab therapy in ulcerative colitis is associated with alterations in immune cell-cell communications. Inflammation Bowel Dis. (2023) 29:1602–12. doi: 10.1093/ibd/izad084. PMID: 37235748 PMC10547234

[52] van Unen V Ouboter LF Li N Schreurs M Abdelaal T Kooy-Winkelaar Y . Identification of a disease-associated network of intestinal immune cells in treatment-naive inflammatory bowel disease. Front Immunol. (2022) 13:893803. doi: 10.3389/fimmu.2022.893803. PMID: 35812429 PMC9260579

[53] Venkat S Rusbuldt J Richards D Freeman T Richmond C Mortensen JH . Serum collagen biomarkers are reflective of tissue specific fibroblasts associated with ulcerative colitis activity and treatment response to ustekinumab. United Eur Gastroenterol J. (2025) 13:982–96. doi: 10.1002/ueg2.70002. PMID: 39969502 PMC12269729

[54] Mayer AT Holman DR Sood A Tandon U Bhate SS Bodapati S . A tissue atlas of ulcerative colitis revealing evidence of sex-dependent differences in disease-driving inflammatory cell types and resistance to TNF inhibitor therapy. Sci Adv. (2023) 9:eadd1166. doi: 10.1126/sciadv.add1166. PMID: 36662860 PMC9858501

[55] Holman D Rogalla S Rogalla L . OP23 neutrophil niches and therapy resistance in ulcerative colitis: Spatial atlas insights. J Crohn's Colitis. (2025) 19:i45–6. doi: 10.1093/ecco-jcc/jjae190.0023. PMID: 41835620

[56] Kim SW Na K Oh SJ Lee CK . OP21 spatial transcriptomics of pre-treatment biopsies revealing molecular maturation state and chronic crypt damage, reflecting histological severity, as predictors of primary responsiveness to TNF-α inhibitors in bio-naive ulcerative colitis patients. J Crohn's Colitis. (2024) 18:i38–9. doi: 10.1093/ecco-jcc/jjad212.0021. PMID: 41835620

[57] Salvador-Martín S Kaczmarczyk B Álvarez R Navas-López VM Gallego-Fernández C Moreno-Álvarez A . Whole transcription profile of responders to anti-TNF drugs in pediatric inflammatory bowel disease. Pharmaceutics. (2021) 13:77. doi: 10.3390/pharmaceutics13010077. PMID: 33429950 PMC7830359

[58] Raine T Vaja S Subramanian S Brezina B Probert CS Steel A . OP33 results of a randomised controlled trial to evaluate interleukin 1 blockade with anakinra in patients with acute severe ulcerative colitis (IASO). J Crohn's Colitis. (2023) 17:i43–6. doi: 10.1093/ecco-jcc/jjac190.0033. PMID: 41835620

[59] Zhang J Maddux R Petersen A Mehandru S Stauffer W Baichwal V . P172 spatial transcriptomics characterization of S1P pathway genes in ulcerative colitis and Crohn’s disease colon tissues. J Crohn's Colitis. (2024) 18:i478. doi: 10.1093/ecco-jcc/jjad212.0302. PMID: 41835620

[60] Turner D Ricciuto A Lewis A D'Amico F Dhaliwal J Griffiths AM . STRIDE-II: An update on the selecting therapeutic targets in inflammatory bowel disease (STRIDE) initiative of the International Organization for the Study of IBD (IOIBD): Determining therapeutic goals for treat-to-target strategies in IBD. Gastroenterology. (2021) 160:1570–83. doi: 10.1053/j.gastro.2020.12.031. PMID: 33359090

[61] Borrelli C Gurtner A Arnold IC Moor AE . Stress-free single-cell transcriptomic profiling and functional genomics of murine eosinophils. Nat Protoc. (2024) 19:1679–709. doi: 10.1038/s41596-024-00967-3. PMID: 38504138

[62] Salcher S Heidegger I Untergasser G Fotakis G Scheiber A Martowicz A . Comparative analysis of 10X Chromium vs. BD Rhapsody whole transcriptome single-cell sequencing technologies in complex human tissues. Heliyon. (2024) 10:e28358. doi: 10.1016/j.heliyon.2024.e28358. PMID: 38689972 PMC11059509

[63] Gao C Zhang M Chen L . The comparison of two single-cell sequencing platforms: BD Rhapsody and 10x Genomics Chromium. Curr Genomics. (2020) 21:602–9. doi: 10.2174/1389202921999200625220812. PMID: 33414681 PMC7770630

[64] Williams CG Lee HJ Asatsuma T Vento-Tormo R Haque A . An introduction to spatial transcriptomics for biomedical research. Genome Med. (2022) 14:68. doi: 10.1186/s13073-022-01075-1. PMID: 35761361 PMC9238181

[65] Zilbauer M James KR Kaur M Pott S Li Z Burger A . A roadmap for the human gut cell atlas. Nat Rev Gastroenterol Hepatol. (2023) 20:597–614. doi: 10.1038/s41575-023-00784-1. PMID: 37258747 PMC10527367

[66] Lee AJ Cahill R Abbasi-Asl R . Machine learning for uncovering biological insights in spatial transcriptomics data. ArXiv. (2023) arXiv:2303.16725v1. doi: 10.1007/978-3-031-01526-7_4. PMID: 41836790

[67] Chelebian E Avenel C Wählby C . Combining spatial transcriptomics with tissue morphology. Nat Commun. (2025) 16:4452. doi: 10.1038/s41467-025-58989-8. PMID: 40360467 PMC12075478

[68] Jia G He P Dai T Goh D Wang J Sun M . Spatial immune scoring system predicts hepatocellular carcinoma recurrence. Nature. (2025) 640:1031–41. doi: 10.1038/s41586-025-08668-x. PMID: 40074893

[69] Lyu D Kou G Li S Li L Li B Zhou R . Digital spatial profiling reveals functional shift of enterochromaffin cell in patients with ulcerative colitis. Front Cell Dev Biol. (2022) 10:841090. doi: 10.3389/fcell.2022.841090. PMID: 35465329 PMC9023741

