# Supplementary material for: Understanding heterogeneity in the pathogenesis and drug responses of ulcerative colitis through single-cell and spatial transcriptomics
Source: Front Immunol. 2026 Mar 31;17:1794207. doi: 10.3389/fimmu.2026.1794207 (PMC13076525; doi:10.3389/fimmu.2026.1794207)
Supplement: Supplementary file 1 [file DataSheet1.docx]

Supplementary Figure 1. PRISMA diagram for study selection

**Included**

References from other sources **(n = 30)**

Citation searching (n =11)

Grey literature (n = 13)

Conference abstract screening (n=6)

Studies screened **(n = 487)**

Studies assessed for eligibility **(n = 95)**

References removed **(n = 39)**

Duplicates identified manually (n = 8)

Duplicates identified by Covidence (n = 31)

Studies excluded **(n = 392)**

Studies excluded **(n = 66)**

Wrong outcomes (n = 4)

Wrong methodology (n = 38)

Wrong study design (n = 17)

Wrong patient population (n = 7)

Studies included in review **(n = 29)**

**Screening**

Studies from databases/registers **(n = 526)**

PubMed (n = 445)

Embase (n = 51)

**Identification**
