# Supplementary material for: Understanding heterogeneity in the pathogenesis and drug responses of ulcerative colitis through single-cell and spatial transcriptomics
Source: Front Immunol. 2026 Mar 31;17:1794207. doi: 10.3389/fimmu.2026.1794207 (PMC13076525; doi:10.3389/fimmu.2026.1794207)
Supplement: Supplementary file 2 [file Table1.docx]

Supplementary Table 1. Customised risk of bias tool

| Domain | Signalling Questions | Response Options |
| --- | --- | --- |
| Confounding | • Did the study account for biological confounders (e.g. disease severity, patient age, treatment history)? • Did the study account for technical confounders (e.g. batch effects, differences in sample collection and preparation)? | Yes / Probably Yes / Probably No / No / No information |
| Classification of Disease | • Were UC samples consistently taken from the same location? • Were healthy controls matched for location? • When studies included 'IBD' patients, were Crohn’s disease and UC reported separately? • For treatment-response studies, was treatment clearly defined and consistently applied (e.g. allowing 6 months of treatment before defining failure per STRIDE II)? | Yes / Probably Yes / Probably No / No / No information |
| Selection of Participants / Cells | • Were captured cells representative of the colonic mucosa (epithelial, stromal, immune)? • Was there evidence of cell capture bias, dropout events, or cell viability issues? | Yes / Probably Yes / Probably No / No / No information |
| Deviation from Intended Protocol | • Was the experimental protocol appropriately reported? • Were any deviations documented and accounted for (e.g. via batch correction methods)? | Yes / Probably Yes / Probably No / No / No information |
| Missing Data | • Did the study report proportions of cells or regions excluded due to QC? • Were missing data handled appropriately? | Yes / Probably Yes / Probably No / No / No information |
| Bias in Measurement of Outcomes | • Was there experimental validation of key findings (e.g. qPCR, in situ hybridisation, immunofluorescence)? • Was there consistency in defining outcomes (e.g. DE thresholds, clustering methods)? | Yes / Probably Yes / Probably No / No / No information |
| Bias in Selection of Reported Results | • Did the study report all DEGs? • Were spatial regions or cellular neighbourhoods equally considered when reporting? • Was there transparency in bioinformatics pipelines? | Yes / Probably Yes / Probably No / No / No information |
