# Supplementary material for: Understanding heterogeneity in the pathogenesis and drug responses of ulcerative colitis through single-cell and spatial transcriptomics
Source: Front Immunol. 2026 Mar 31;17:1794207. doi: 10.3389/fimmu.2026.1794207 (PMC13076525; doi:10.3389/fimmu.2026.1794207)
Supplement: Supplementary file 3 [file Table2.docx]

Supplementary Table 2. Summary of the Spatial Technologies and their key characteristics

| Spatial Technology | Target | Chemistry | Throughput | Resolution |
| --- | --- | --- | --- | --- |
| Spatial Transcriptomics – Sequencing based | | | | |
| 10x Genomics Visium | RNA | PolyA capture on barcoded slide | Whole transcriptome (~20,000 genes) | 55 μm spots (multi-cellular) |
| 10x Genomics Visium HD | RNA | PolyA capture via probe transfer | Whole transcriptome | ~2 µm barcoded squares (subcellular) |
| BGI Stereo-seq | RNA | PolyA capture on DNA nanoball arrays | Whole transcriptome | ~500 nm (subcellular) |
| Curio Bioscience Curio Seeker | RNA | Capture on high-density barcoded arrays | Whole transcriptome | 5–10 μm (near single-cell) |
| Nanostring GeoMx DSP | Protein / RNA | Barcoded antibody or probe hybridisation with optical readout | Whole transcriptome plus  Up to ~100 proteins | Region-specific (not single-cell) |
| Spatial Transcriptomics – Imaging based | | | | |
| Nanostring CosMx SMI | RNA | Multiplexed probe hybridisation + imaging | Up to 1,000 genes | Single-cell/subcellular |
| 10x Genomics Xenium | RNA | Barcoded probe hybridisation + fluorescence imaging | 5,000 genes plus ~400 in a targeted panel | Subcellular |
| Spatial Proteomics – Imaging based | | | | |
| Akoya Biosciences CODEX | Protein | DNA-barcoded antibodies with iterative fluorescent imaging | ~60–100 proteins | Single-cell/subcellular |
| IonPath MIBI | Protein | Metal-tagged antibodies with secondary ion mass spectrometry | ~40 markers | Single-cell/subcellular |
| Spatial Proteomics – Mass Spectrometry based | | | | |
| MALDI Imaging MS | Protein / Peptides | Label-free mass spectrometry | A few hundred to over one thousand proteins | 10–50 μm (multi-cellular) |
| Fluidigm Hyperion | Protein | Metal-tagged antibodies detected by mass cytometry | ~40 markers | Single-cell |
